# Landscape of gene transposition–duplication within the Brassicaceae family

**DOI:** 10.1093/dnares/dsy035

**Published:** 2018-10-30

**Authors:** Dong-Ha Oh, Maheshi Dassanayake

**Affiliations:** Department of Biological Sciences, Louisiana State University, Baton Rouge, LA, USA

**Keywords:** gene transposition, gene duplication, synteny, co-linearity, OrthNet

## Abstract

We developed the CLfinder-OrthNet pipeline that detects co-linearity among multiple closely related genomes, finds orthologous gene groups, and encodes the evolutionary history of each orthologue group into a representative network (OrthNet). Using a search based on network topology, we identified 1,394 OrthNets that included gene transposition–duplication (*tr–d*) events, out of 17,432 identified in six Brassicaceae genomes. Occurrences of *tr–d* shared by subsets of Brassicaceae genomes mirrored the divergence times between the genomes and their repeat contents. The majority of *tr–d* events resulted in truncated open reading frames (ORFs) in the duplicated loci. However, the duplicates with complete ORFs were significantly more frequent than expected from random events. These were derived from older *tr–d* events and had a higher chance of being expressed. We also found an enrichment of *tr–d* events with complete loss of intergenic sequence conservation between the original and duplicated loci. Finally, we identified *tr–d* events uniquely found in two extremophytes among the six Brassicaceae genomes, including *tr–d* of *SALT TOLERANCE 32* and *ZINC TRANSPORTER 3* that relate to their adaptive evolution. CLfinder-OrthNet provides a flexible toolkit to compare gene order, visualize evolutionary paths among orthologues as networks, and identify gene loci that share an evolutionary history.

## 1. Introduction

Co-linearity among closely related genomes erodes over time due to the accumulation of mutations including gene duplication, deletion, and transposition.^1^ Gene duplication affects gene dosage, which may lead to divergence of expression and functions among duplicates.^2^^,^^3^ Gene transposition events modify expression strength and tissue-specificity through changes in regulatory sequences,^4^^,^^5^ local epigenetic environment,^6^ and proximity to enhancers and chromatin structural contexts.^7–9^ Such events have been associated with variation in copy numbers of genes and transcripts,^5^^,^^10^ as well as localization^11^ and functions^12–14^ of encoded proteins among orthologous genes. A large number of studies have reported examples of gene level duplications and transpositions as key underlying sources for adaptations to specific environments or speciation.^15–21^

Comparative analysis of co-linearity and its erosion identifies modes of gene duplications^22^^,^^23^ and traces the origin of genes or gene families and their evolutionary history.^24–26^ Such comparative analyses are facilitated by *de novo* assembled genomes released at unprecedented rates today,^27–30^ enabling detection of gene gain and loss as well as duplication and transposition among closely related taxa. These resources also call for novel methods and tools for systematic comparative analysis of genomes.

A number of tools are available for identification of gene blocks or large genomic regions co-linear among multiple genomes.^31–33^ Another set of tools can identify orthologues in related genomes for a gene of interest and visualize synteny and evolutionary events, such as gene duplication and transposition, associated with them.^34–38^ However, currently we do not have a method that can retrieve all orthologue loci within multiple genomes that have likely undergone the same set of evolutionary events without a prior assignment of a gene of interest.

To address this, we introduce the CLfinder-OrthNet pipeline, which identifies co-linearity (CL) in the gene order among multiple genomes, identify ‘orthologue groups’ based on co-linearity, and encodes genes in each orthologue group as a network of orthologues (OrthNet). Each orthologue group includes orthologues and paralogues likely derived from a single ancestral locus in multiple target genomes. All evolutionary events in each orthologue group, such as gene duplication, deletion, transposition, and any combination of them, in addition to gene synteny conservation, are captured as the topology of an OrthNet. Our pipeline enables detection of all orthologue groups from multiple genome that seemingly underwent the same evolutionary events, by searching OrthNets essentially based on their topologies.

As a proof-of-concept, we applied the CLfinder-OrthNet pipeline to six Brassicaceae (crucifer) genomes that share the same whole genome duplication history.^39^ The six Brassicaceae genomes included the model species *Arabidopsis thaliana*^40^ and extremophytes *Schrenkiella parvula*^41^ and *Eutrema salsugineum*,^42^^,^^43^ the two most salt-tolerant Brassicaceae species so far tested.^44^*S. parvula* and *E. salsugineum* are biogeographicaly seperated and represent taxa adapted to multi-ion salt strsses in soils near a hypersaline lake in central Anatolia^5^ and combined salt and freezing stresses in salt pans of high latitude regions in the northern hemisphere,^45^^,^^46^ respectively. These two extremophytes provide optimal models for comparative analyses to study plant adaptations to environmental challenges.^47^^,^^48^

The CLfinder-OrthNet pipeline detects any combination of gene synteny conservation, duplications, deletions, and transpositions. For this study, we focused on the relatively under-studied transposition–duplication (*tr–d*) events within the six Brassicaceae genomes. A *tr–d* is an event where a single non-transposon gene is duplicated and relocated to a position where all evidence of common ancestry (i.e. synteny) is lost, i.e. ‘Duplication mode IV’ as defined by Freeling.^22^ A *tr–d* event results in variations in both copy numbers and co-linearity and, unlike other types of gene duplications, requires a systematic comparison of multiple genomes for detection.^22^^,^^49^ Our pipeline identified *tr–d* events unique to a genome or shared by any subset of the six Brassicaceae genomes, as well as the original donor and duplicate loci in each *tr–d* event including loci with truncated coding regions. Using this pipeline, we aim to identify the landscape of lineage-specific and shared *tr–d* events among the target genomes; test whether there is a signature of selective retention among lineage-specific *tr–d* events; and characterize *tr–d* events unique to the extremophyte genomes, which may have contributed to their adaptive evolution.

## 2. Materials and methods

### 2.1. Genome and gene models

**Figure 1.**
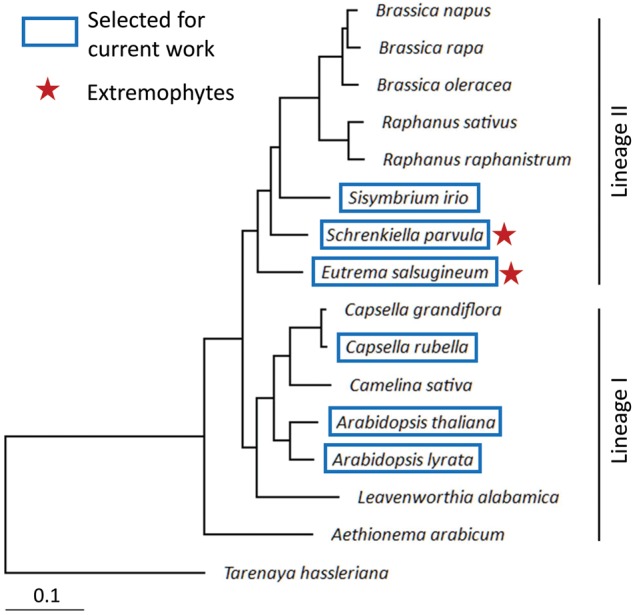
A comparative genomics framework including the two extremophyte/halophyte crucifers, *S. parvula* and *E. salsugineum*. Boxes and stars indicate the six Brassicaceae species selected for this work and halophytes, respectively. The tree was based on an alignment of 14,614 homologous gene clusters, as described in Materials and Methods.

We obtained genome annotations of *Arabidopsis lyrata* (Aly, version 1.0), *A. thaliana* (Ath, v. ‘TAIR10’), *Capsella rubella* (Cru, v. 1.0), and *E. salsugineum* (Esa, v. 1.0) from Phytozome v. 11 (http://genome.jgi.doe.gov/ (10 October 2018, date last accessed)), while genomes of *Sisymbrium irio* (Sir, v. 0.2; CoGE genome id 19579) and *S. parvula* (v. 2.0) were downloaded from CoGE (https://genomevolution.org/coge/ (10 October 2018, date last accessed)) and thellungiella.org (http://thellungiella.org/data/ (10 October 2018, date last accessed)), respectively. For *S. irio* annotation, we used a combination of RepeatMasker, a Basic Local Alignment Search Tool for Nucleotides (BLASTN)^50^ search vs Repbase v. 20170127 (http://www.girinst.org/repbase/ (10 October 2018, date last accessed)), and OrthoMCL^51^ to further filter out gene models most likely unannotated transposable elements (TEs). This step was added to remove the large number of repetitive lineage-specific genes (34.9% of 49,956 gene models), many of them showing sequence similarities with known TEs, in the *S. irio* genome annotation. To generate a species tree of Brassicaceae genomes including the six target species (Fig. 1), we used Agalma^52^ which built a maximum likelihood tree based on 14,614 alignments of homologous protein-coding gene clusters. Each cluster contained sequences from more than four crucifer genomes. We listed in Supplementary Table S1 additional genome resources used for the Brassicaceae species tree.

For the analysis of co-linearity erosion (Supplementary Text S1 and Fig. S1), we compared all protein-coding gene loci in five crucifer genomes to those in *A. thaliana* using BLASTN. Four degenerate site (4d) substitution rate distributions were analysed for all reciprocal BLASTN pairs of protein-coding genes between the five crucifer genomes and *A. thaliana*, using codeml^53^ and custom scripts. TE contents were determined using RepeatMasker v. 4.0.7 and RepeatModeler v. 1.0.8 (http://www.repeatmasker.org/ (10 October 2018, date last accessed)), for all six target species.

### 2.2. Detecting co-linearity in gene orders among Brassicaceae genomes

**Figure 2.**
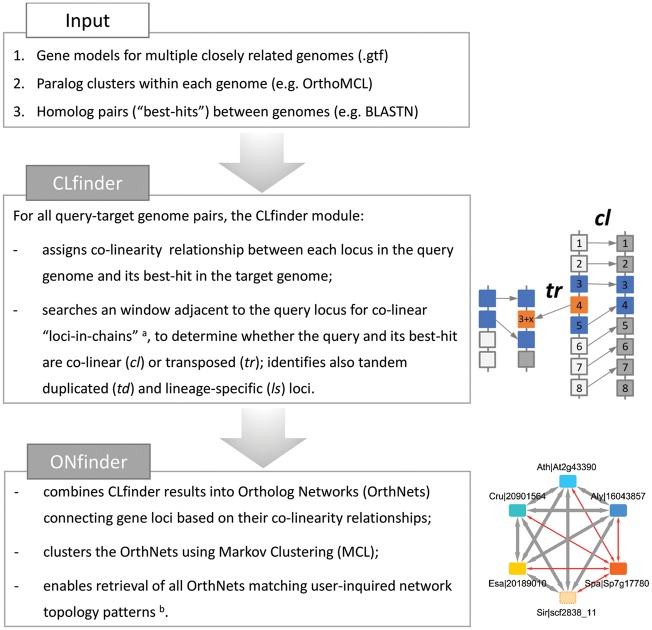
The CLfinder-OrthNet pipeline consists of two modules, CLfinder (Co-Linearity finder) and ONfinder (OrthNet finder). In the cartoon next to the box for the CLfinder module, blue squares indicate gene loci with disrupted co-linearity due to mutations and indels in neighbouring loci, while orange squares are those transposed and lost the synteny. See Supplementary Fig. S1 for details. Next to the box for the ONfinder module is an exemplary OrthNet. (A) For the detailed method to determine co-linearity (CL) relationship between the query loci and their most homologous counterpart (‘best-hits’) in the target genome, see Materials and Methods (2.2) and Figs S2 and S3. CLfinder output for the six crucifer species highlighted in Fig. 1 are summarized in Table 1, with the full results given as Supplementary Dataset S1. (B) See Figures 3–6, for examples of OrthNets with different evolutionary histories represented as different network topologies, e.g. transposition (*tr*) and transposition–duplication (*tr–d*) unique to each species or a group of species.

We developed the CLfinder process to scan the order of homologous gene loci between a pair of genomes and detect co-linearity among them. The CLfinder module performs this process automatically for all pairs among multiple closely related genomes. The CLfinder uses three inputs (Fig. 2 and Supplementary Fig. S2), for which we used only primary transcript models for protein-coding gene loci. **Input 1**: A custom script (‘parse_gtf.py’) parsed genome annotation in Genomic Transfer Format (GTF). **Input 2**: Intra-species paralogous loci detected by clustering primary protein-coding gene model sequences within each species using OrthoMCL,^51^ as previously described in ref. 5. **Input 3:** Results of pairwise reciprocal BLASTN (*e* < 10^−5^) between all primary protein-coding gene model ORFs to obtain the ten most similar BLASTN hits (‘best-hits’) in the target species, for each gene model in the query species.

The sensitivity and stringency of the CLfinder process are adjustable based on three user-defined parameters, the window size (*W*), the number of co-linear loci-in-chain threshold (*N*), and the maximum gap allowed between co-linear loci-in chain (*G*) (Supplementary Fig. S2). For each query locus (from Input 1), CLfinder scans and counts the number of ‘loci-in-chain’, whose best-hits (from Input 3) are separated by the same or less than *G* from the best-hit of their immediate neighbours. The scan starts from the query locus and moves towards both up- and downstream of the query genome. If more than *N* loci-in-chain, including the query locus, are found within a window size of 2 × *W* (i.e. *W* to the up- and downstream), the query locus is declared co-linear (*cl*) with its best-hit locus in the target species. When loci-in-chain were found only towards one direction, the query-target best-hit pair is considered to represent an end of a co-linear genome segment derived from inversions, indels, and segmental duplications involving multiple gene loci, and designated as ‘*cl_end*’. We excluded lineage-specific (*ls*) loci that did not have a best-hit in the target species from the search for co-linear loci-in-chain. Tandem duplicated (*td*) loci, defined as adjacent loci separated by the same or less than *T* loci (Supplementary Fig. S2) and in the same paralogue cluster defined in Input 2, were counted as a single locus during the co-linearity search step. A query locus neither co-linear (*cl*) nor lineage-specific (*ls*) was considered to be transposed (*tr*). If the query locus is in a genome assembly scaffold that contains less than *W* loci, the pipeline will mark the query locus as not-determined (*nd*) instead of transposed (*tr*). Segmental rearrangements such as local inversion are identified based on loci-in-chain detected exclusively in either up- or down-stream. In such cases, CLfinder declares the query and its best-hit as *cl_end*.

For the analysis of six crucifer genomes, CLfinder parameters (*W* = 20, *N* = 3, *G* = 20, and *T* = 4) were decided based on the distribution of protein-coding gene locus content in the scaffolds of the most fragmented genome and the results from the analysis of co-linearity erosion. The window size (*W*) was chosen based on the most fragmented *S. irio* genome, where >63% of total genes were in scaffolds that contained at least 20 gene loci. Maximum gap between co-linear loci-in-chain (*G* = 20) was decided to distinguish gene order displacements likely caused by local indels from those by transpositions, based on the result of co-linearity erosion analysis (Supplementary Text S1 and Fig. S1). With *W* and *N* parameters set, we tested multiple number of co-linear loci-in-chain threshold (*N*) parameters to detect co-linear genes between simulated genomes of 27,000 gene loci, assuming a complete random shuffling and lack of a common ancestor between genomes. With *N* = 3, after 10,000 simulations comparing randomly shuffled simulated genomes, CLfinder found on average only 39.43 ± 9.85 genes (0.15 ± 0.04% of the simulated genome) as false-positive co-linear genes, indicating that the selected parameters can effectively rule out co-linearity by chance and without a common ancestry (Supplementary Table S2). We determined the maximum tandem duplication distance (*T* = 4) to enable detection of nested tandem duplication events while filtering out tandem duplications with too many unrelated genes inserted in between. The CLfinder process (Supplementary Fig. S3) was performed for all possible query-target pairs for the six crucifer species using a wrapper script in the CLfinder module (Supplementary Fig. S2, ‘CLfinder_multi.py’). The results are combined into a single table and given as Supplementary Dataset S1.

### 2.3. Construction of OrthNets for Brassicaceae genomes

The second module of the pipeline, ONfinder (OrthNet finder), combines the output of the CLfinder module and produces networks of orthologous loci (OrthNet). OrthNets represent primary protein-coding gene loci from all species (nodes) connected with their best-hits with directional edges, and the co-linearity relationship (i.e. either *cl*, *tr*, or *nd*) between them as the edge property. In addition, OrthNets include tandem duplicated paralogues connected with undirected edges (*td*) among themselves. The ONfinder module also compares the ORF size of a node to the median ORF size of all neighbouring nodes to detect truncated ORFs (i.e. of sizes less than 40 and 80% of the median ORF size) in each OrthNet.

ONfinder first clusters all nodes from all species connected with an edge (Supplementary Fig. S2, ‘create_OrthNet.py’). This process often results in an aggregation of multiple loci connected with unidirectional edges in a single large OrthNet, due to duplicated paralogues, lineage-specific deletions, and partial gene models that lead to non-reciprocal best-hit pairs. We employed two methods to alleviate this issue. First, for all unidirectional edges from node A to node B, ONfinder searches for an alternative best-hit for B, among the list of the ten best-hit candidates, that makes A and B a reciprocal best-hit pair (Supplementary Fig. S2, ‘update_BestHitPairs.py’). The best-hit candidates were obtained by running BLASTN with the ‘-max_target_seqs 10’ option when creating the Input 2 for the CLfinder module. The ONfinder module records how many best-hit candidates it tested before identifying a reciprocal best-hit pair (Supplmentary Dataset S1, legend). Only when a reciprocal best-hit does not exist among all ten alternatives, the OrthNet will have a unidirectional edge. Second, each OrthNet is subjected to further clustering to finer OrthNets using Markov Chain Clustering (MCL),^54^ with an inflation parameter (1.2) and a higher edge weight given in the order of *td* (1.5), reciprocal *cl* (1.2), unidirectional *cl* (0.6), reciprocal *tr* (0.5), and unidirectional *tr* (0.25) edges (default edge weight values used in this study are in parentheses). Edge weights for the MCL process were decided as detailed in Supplementary Text S2 and Fig. S4, with the following aims: (i) to separate networks of out-paralogues derived from multiple loci duplicated prior to the divergence of the six target genomes (as exemplified in Supplementary Fig. S4), while (ii) keeping paralogues that underwent tandem duplication (*td*), transposition–duplication (*tr–d*), and combinations of *td* and *tr–d*, together with the core set of co-linear orthologues in the same OrthNet. Once an OrthNet was separated into multiple smaller OrthNets, any edge removed by MCL was replaced by an alternative edge connecting nodes within each of new OrthNets, by searching for an alternative best-hit pair among the list of ten best-hit candidates (Supplementary Fig. S4C). The ONfinder module records the number of best-hit candidates tested before the final edge was decided (Supplementary Dataset S1). The MCL process effectively removed spurious unidirectional edges that connect nodes from different loci (Supplementary Fig. S2. ‘update_OrthNet_afterMCL.py’, see also Supplementary Text S2 and Fig. S4 for detailed discussion and an example). The final network units (OrthNets) were converted to the Simple Interaction File (SIF) format for visualization using Cytoscape (http://cytoscape.org/ (10 October 2018, date last accessed)). All OrthNets derived from this analysis are included as archived SIFs (Supplementary Dataset S2).

### 2.4. Searching OrthNets that share an evolutionary history

While CLfinder and ONfinder can operate independently, when used in a pipeline, the ONfinder module adds the results of its analysis to the CLfinder result for each locus (Supplementary Dataset S1). For each gene locus that belonged to an OrthNet, ONfinder adds the unique ID of the OrthNet that includes the locus, number of nodes derived from each genome (i.e. gene copy numbers) in the OrthNet, and the edge types connecting the locus to its best-hit nodes in other genomes (Supplementary Dataset S1). This information enabled identifying OrthNets with the same or similar topologies that represent the same set of evolutionary events.

**Figure 3.**
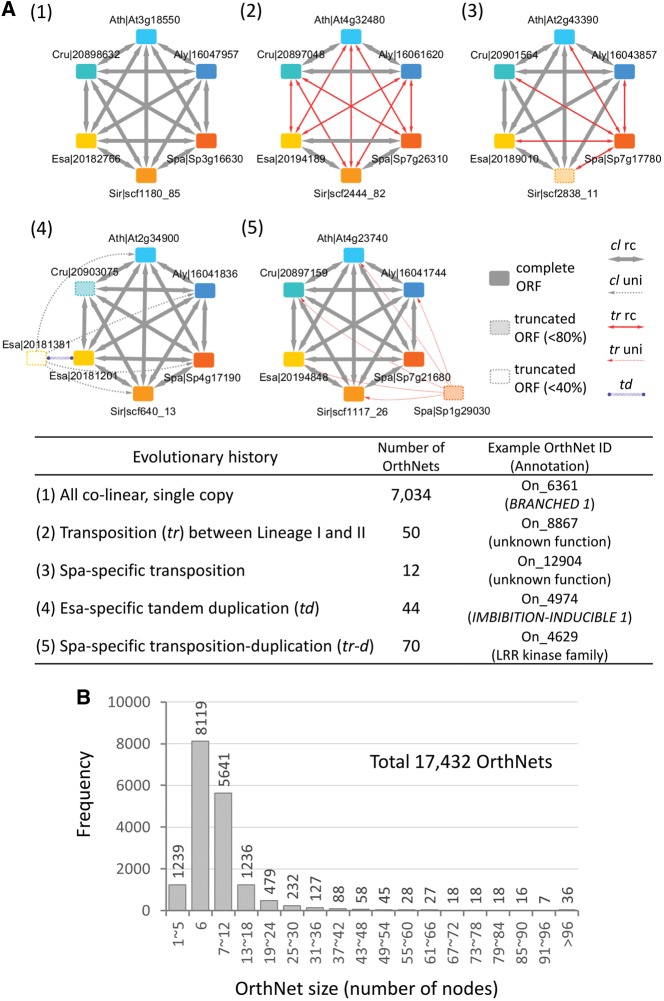
The ONfinder module encodes the evolutionary history of an orthologous gene group into an OrthNet. (A) OrthNet examples representing five different evolutionary histories of orthologous gene groups derived from the six Brassicaceae genomes (Fig. 1, highlighted with boxes). Nodes are colour-coded according to the species. Transparent nodes with dashed borders indicate loci with truncated ORFs, i.e. ORF sizes smaller than either 80% or 50% compared with the median ORF size of nodes they are connected to. Edges show properties either co-linear (*cl*) or transposed (*tr*), reciprocally (rc) or unidirectionally (uni). Tandem duplicated (*td*) paralogues are connected by undirected edges. The lower panel shows the ID and annotation for representative OrthNets, as well as the number of OrthNets representing the same evolutionary history among 17,432 OrthNets identified for the six Brassicaceae genomes. (B) A histogram showing the size distribution of OrthNets.

The ONfinder module offers two methods to search OrthNets (Supplementary Fig. S2). To systematically identify OrthNets including *tr–d* events uniquely found in subsets of the six crucifer genomes, we searched for OrthNets that (i) showed a node copy number pattern consistent with duplications specific to the subset and (ii) contain nodes that are connected to all of their orthologous neighbours through unidirectional *tr* edges. We used the ‘search_OrthNet_pattern.py’ script (Supplementary Fig. S2) and searched for node copy number and edge property patterns as listed in Supplementary Table S3, in the CLfinder-OrthNet output (Supplementary Dataset S1). For evolutionary history patterns presented in Fig. 3A, the second script, ‘search_OrthNet_topology.py’ (Supplementary Fig. S2), found all OrthNets with either exactly the same or similar topologies with the query OrthNet.

### 2.5. Analysis of transposition–duplication (*tr–d*) events

Within an OrthNet with a *tr–d* event, the *tr–d* donor or ‘CL copy’ was the node connected to orthologous nodes with the most *cl* edges, while the remaining were *tr–d* acceptors or ‘Tr copies’. When multiple CL copies existed due to tandem duplication, we used the one with the longest ORF as the representing CL copy. Homologous Genome Segments (HGSs) were detected between the gene and ±5 kb intergenic regions of the CL copy and each of Tr copies, using LASTZ with chaining as previously described in ref. 5. A *tr–d* event was ‘complete’ if the HGS included the entire CL copy gene. A ‘gene-only’ *tr–d* was defined as a complete *tr–d* event with the size of HGS less than 120% of the coding region of the CL copy gene. We determined the expected occurrences of complete and gene-only *tr–d* by random shuffling and overlapping of HGSs and CL copy genes. Specifically, we assumed a random HGS selected among the observed HGS length distribution occurred at a random position in the vicinity of a CL copy gene, with a minimum 60 bps overlap between the genic region and a HGS. The distribution of such occurrences from 10,000 iterations was fitted to a normal distribution to calculate the *P*-value of the observed occurrence, using the fitdistr function in R MASS package (https://cran.r-project.org/web/packages/MASS (10 October 2018, date last accessed)).

The four degenerate site (4d) substitution rates were calculated for all CL and Tr copy pairs where the Tr copy contained a complete ORF, using codeml^53^ and a custom script (Supplementary Fig. S2, ‘pairwiseKs_by_codeml.py’). All custom scripts used in this study are available at the CLfinder-OrthNet GitHub page (https://github.com/ohdongha/CL_finder). To determine Tr copies with expression evidence, we used RNA-seq data for leaf and root tissues obtained from Wang et al.^13^ for *A. lyrata*, *A. thaliana*, and *C. rubella*, and Oh et al.^5^ for *A. thaliana* and *S. parvula* as well as samples prepared for this study (for *E. salsugineum*, BioProject ID PRJNA63667) as previously described in Oh et al.^5^ FPKM values of representative transcript models were estimated using Stringtie (v. 1.3.1c) with the ‘-e’ option,^55^ after RNAseq reads were aligned to the genome using HISAT2 (v. 2.0.5).^55^

## 3. Results

### 3.1. Patterns of co-linearity erosion within the six Brassicaceae genomes

We selected a set of six genomes with the same whole genome duplication history sampled from the Brassicaceae Lineages I and II for this study (Fig. 1). This set includes the model plant *A. thaliana* (Ath) and its relatives in Lineage I, *A. lyrata* (Aly) and *Capsella rubella* (Cru), as well as *Sisymbrium irio* (Sir) and the two extremophytes, *E. salsugineum* (Esa) and *S. parvula* (Spa), in Lineage II. Figure 1 shows the phylogenetic relationships of the target species with other published genomes in Brassicaceae based on amino acid sequence alignments of 14,614 homolog clusters (see Materials and Methods, 2.1).

Before applying the CLfinder-OrthNet pipeline, we analysed the degree of co-linearity erosion among the target Brassicaceae genomes by comparing gene orders, as detailed in Supplementary Text S1 and Fig. S1. Our analysis revealed that two-thirds of genes identified as non-transposable element (non-TE) and non-lineage-specific (non-LS) genes in the Brassicaceae genomes showed a conservation of gene order with their immediate neighbours when compared with the genome of *A. thaliana* (Supplementary Fig. S1C, *d_n_*_,__*n*__+1_ ≤1). The proportion of non-TE and non-LS gene loci showing a proximal (Supplementary Fig. S1C, *d_n_*_,__*n*__+1_ = 2–20) and distal (Supplementary Fig. S1C, *d_n_*_,__*n*__+1_ >20 and ‘Diff Chr’) gene order displacement, compared with their immediate neighbours, was correlated with the divergence time between genomes and their TE contents, respectively (Supplementary Fig. S1D). This suggested two different models for co-linearity erosion, as summarized in Supplementary Fig. S1E. The proximal gene order displacements were likely resulted from mutations and indels accumulated in the neighbouring loci over time (Supplementary Fig. S1A, blue loci). In this model, larger gene order displacements requiring multiple mutations in the neighbouring gene loci are rarer, which explains the rapid decline of genes in Supplementary Fig. S1B as *d_n_*_,_*_n+_*_1_ increases from 1, i.e. perfect co-linearity, to larger values (Supplementary Fig. S1E, blue line and arrow). The second model involves transposition of mostly single locus (Supplementary Fig. S1A, orange loci), which may have been initiated by the presence of repetitive sequences and TE activities. Transposition based on mechanisms such as the repair of double strand breaks by non-homologous end-joining (DSB-NHEJ)^1^ can occur ubiquitously between any pair of genomic locations with a frequency lower than that of point mutations (Supplementary Fig. S1E, orange line and arrow). Genes showing larger *d_n_*_,_*_n+_*_1_ with their neighbours in both directions are most likely transposed via the second model. We used the result from co-linearity erosion (Supplementary Fig. S1B) to determine parameters suitable for detecting co-linear loci between a pair of genomes, as detailed in Materials and Methods (2.2) and Supplementary Text S1.

### 3.2. Development of the CLfinder-OrthNet pipeline

Our pipeline consists of two modules: CLfinder and ONfinder (Fig. 2 and Supplementary Fig. S2). The first module, CLfinder, compares all possible pairs of query and target genomes and test whether each homologous gene pair (i.e. ‘best-hit’ pair, Supplementary Text Glossary) is co-linear (Supplementary Figs S2 and S3, and Dataset S1). CLfinder accepts three inputs: representative gene models for all loci in each genome, clusters of paralogues within each genome, and lists of best-hits between all possible query-target genome pairs (Fig. 2 and Supplementary Fig. S2). Users can select the methods and criteria for defining paralogue clusters and best-hit pairs, as well as the sensitivity and stringency for the co-linearity detection by controlling three parameters: the window size (*W*), the number of co-linear loci-in-chain threshold (*N*), and the maximum gap allowed between co-linear loci-in chain (*G*) (see Materials and Methods, 2.2). Based on these parameters, the CLfinder module searches both up- and downstream of each locus in the query genome for ‘loci-in-chain’ based on the order of their best-hits in the target genome, to determine whether a query-target best-hit pair is either co-linear (*cl*), transposed (*tr*), or not able to determine (*nd*) due to the query genome assembly scaffold being too short. When co-linearity was detected only towards one direction, the query-target best-hit pair is considered representing an end of a co-linear genome segment (*cl_end*) derived from inversions, indels, and segmental duplications involving multiple gene loci. A query locus without a best-hit in the target genome is marked lineage-specific (*ls*) (Supplementary Fig. S3).

The second module, ONfinder, combines all pairwise comparisons by CLfinder and encodes co-linearity relationships among orthologues into networks (OrthNets), with gene loci as nodes connected by an edge to their best-hits in other genomes (Figs 2 and 3). Each edge has a property of either co-linear (*cl*), transposed (*tr*), or not determined (*nd*). The *cl* and *tr* edges can be either reciprocal or unidirectional (Fig. 3A, ‘rc’ and ‘uni’, respectively). OrthNets also include tandem duplicated (*td*) paralogues, connected by undirected edges [e.g. panel (4) in Fig. 3A]. ONfinder uses Markov clustering (MCL),^54^ based on edge weights assigned according to edge properties, to divide large networks that are often a result of expanded gene families with a large number of paralogues into smaller clusters likely derived from a single ancestral locus (Supplementary Fig. S4 and Text S2). Each cluster of orthologues, separated by MCL, is given an OrthNet ID and represented as an orthologue network or an OrthNet. Finally, ONfinder can search with a user-defined pattern of orthologue copy numbers, edge characteristics, and network topology, to retrieve all OrthNets sharing a given set of evolutionary events (see Materials and Methods, 2.4). Several selected examples of OrthNets representing different evolutionary histories are shown in Fig. 3A.

### 3.3. Identification of OrthNets among six Brassicaceae genomes

We tested the CLfinder-OrthNet pipeline on the six Brassicaceae genomes using parameters and input files as described in Materials and Methods. The CLfinder module summarizes all reciprocal query-target genome pairwise analyses as exemplified for the six Brassicaceae genomes in Table 1. For simplicity, we considered *cl_end* loci pairs as *cl* in this summary. All query-target genome pairs showed a comparable number of *cl* loci pairs, ranging from 19,015 (Table 1, Sir–Aly) to 24,296 (Table 1, Aly–Ath). The number of *cl* pairs follows the division of the Lineage I (Table 1, Aly, Ath, and Cru) and II (Table 1, Esa, Sir, and Spa), with higher numbers observed between query-target pairs within each Lineage. The number of *tr* loci pairs was proportional to the repeat contents of the query genomes. For example, *A. lyrata* and *E. salsugineum* are the query genomes with the highest content of *tr* pairs (Table 1, Aly and Esa), which correlated with the higher content of repeats in these two genomes than in *A. thaliana*, *C. rubella*, or *S. parvula* genomes [‘TE(%)’ row in Supplementary Table S4]. When *S. irio* was the query, the proportion of *nd* pairs was higher than all other genomes (Table 1, Sir), because it had the most fragmented genome assembly among the six genomes. The entire CLfinder results for all query-target genome pairs are in Supplementary Dataset S1.

**Table 1.** Summary of CLfinder results showing pairwise comparisons among 6 crucifer species

Query species	# Protein-coding genes	CL type^a^	Target species						# *td*^b^ events (# *td* genes)
			Aly	Ath	Cru	Esa	Sir	Spa	
Aly	32,657	*cl*		24,296	23,055	21,416	19,988	21,032	2,163 (5,733)
		*tr*		4,881	5,375	6,668	8,104	6,478	
		*ls*		2,876	3,611	3,954	3,902	4,530	
		*nd*		604	616	619	663	617	
Ath	27,206	*cl*	23,436		22,683	21,187	19,821	20,851	1,747 (4,770)
		*tr*	2,431		2,804	4,032	5,355	4,064	
		*ls*	1,339		1,719	1,987	2,030	2,291	
		*nd*	0		0	0	0	0	
Cru	26,521	*cl*	22,371	22,836		20,906	19,350	20,436	1,752 (4,996)
		*tr*	3,036	2,836		4,338	5,817	4,267	
		*ls*	950	666		1,112	1,154	1,646	
		*nd*	164	183		165	200	172	
Esa	26,351	*cl*	20,384	20,884	20,460		19,699	20,612	1,646 (4,461)
		*tr*	4,465	4,137	4,460		5,431	4,046	
		*ls*	1,452	1,274	1,377		1,146	1,631	
		*nd*	50	56	54		75	62	
Sir	32,524	*cl*	19,015	19,538	19,068	19,728		19,766	1,795 (4,586)
		*tr*	3,062	2,860	2,998	2,722		2,697	
		*ls*	5,520	5,148	5,496	5,054		4,225	
		*nd*	4,927	4,978	4,962	5,020		5,836	
Spa	26,847	*cl*	19,849	20,358	19,934	20,380	19,546		1,242 (3,049)
		*tr*	2,830	2,452	2,718	2,534	4,097		
		*ls*	3,649	3,541	3,688	3,432	2,526		
		*nd*	519	496	507	501	678		

a Co-linear (*cl*), transposed (*tr*), lineage-specific (*ls*), or not determined due to too small genome scaffold (*nd*), with CLfinder parameters {window_size, num_CL_trshld, gap_CL_trshld} = { 20, 3, 20 }.

b Tandem duplication (*td*) detected using the parameter max_TD_loci_dist = 4.

The ONfinder module combined all pairwise CLfinder analyses and developed OrthNets representing the evolutionary history of each set of orthologous loci as the network topology (Fig. 3). For an analysis of N genomes, a perfect polygon (e.g. hexagon in this study) with each of N nodes connected to other nodes by N-1 bidirectional solid grey edges represents a single-copy co-linear orthologous gene in all genomes [Fig. 3A, panel (1)]. We identified a total of 7,034 OrthNets that showed single-copy loci co-linear to each other in all genomes. Panel (2) of Fig. 3A is an example from 50 OrthNets with co-linearity found within each of the Lineages I and II while loci between the two Lineages were transposed, representing a transposition event following the lineage divergence. Panel (3) shows one of the nine OrthNets with only the locus in *S. parvula* transposed compared with all other species. We found 44 OrthNets with the same evolutionary history depicted in panel (4), i.e. *E. salsugineum*-specific tandem duplication, and 86 OrthNets for *S. parvula*-specific transposition–duplication (*tr–d*) events shown in panel (5) of Fig. 3A. ONfinder also compares the ORF size of a node with the median ORF size of all other orthologous nodes to which the node is connected, to identify truncated ORFs [e.g. panels (3), (4), and (5) in Fig. 3A]. We included all OrthNets together with the CLfinder results identified among the six Brassicaceae genomes in Supplementary Dataset S1.

An OrthNet may include a disproportionately large number of duplicated gene loci in specific genomes. For example, an OrthNet showing *A. lyrata*-specific *tr–d* events included 82 nodes representing additional *A. lyrata* transposed–duplicated paralogue copies (Supplementary Fig. S5). Such duplication events, as well as large gene families where exact reciprocal orthologue pairs were hard to identify among multiple paralogues, may result in an OrthNet with a large number of nodes. However, more than 85% of OrthNets contain the same or less than 12 nodes per OrthNet (14,849 out of total 17,432 OrthNets), likely derived from single ancestral loci with duplications restricted in a subset of the six Brassicaceae genomes (Fig. 3B). The size distribution of OrthNets was also comparable with that of orthologous gene clusters detected based on sequence homology by OrthoFinder^56^ (Supplementary Fig. S6A). The majority of OrthNets was matched 1-vs-1 with an OrthoFinder cluster (Supplementary Fig. S6B). In all OrthNet-OrthoFinder cluster pairs, 70.1% total OrthNets contained the set of genes identical to, and additional 12.3% OrthNets showed more than 80% overlap with, their corresponding OrthoFinder clusters (Supplementary Fig. S6C, dashed box). An example of OrthNet different from the orthologous gene cluster detected by OrthoFinder is shown in Supplementary Fig. S4 and Text S2, respectively.

### 3.4. Characterization of lineage-specific and shared *tr–d* events among Brassicaceae genomes

**Figure 4.**
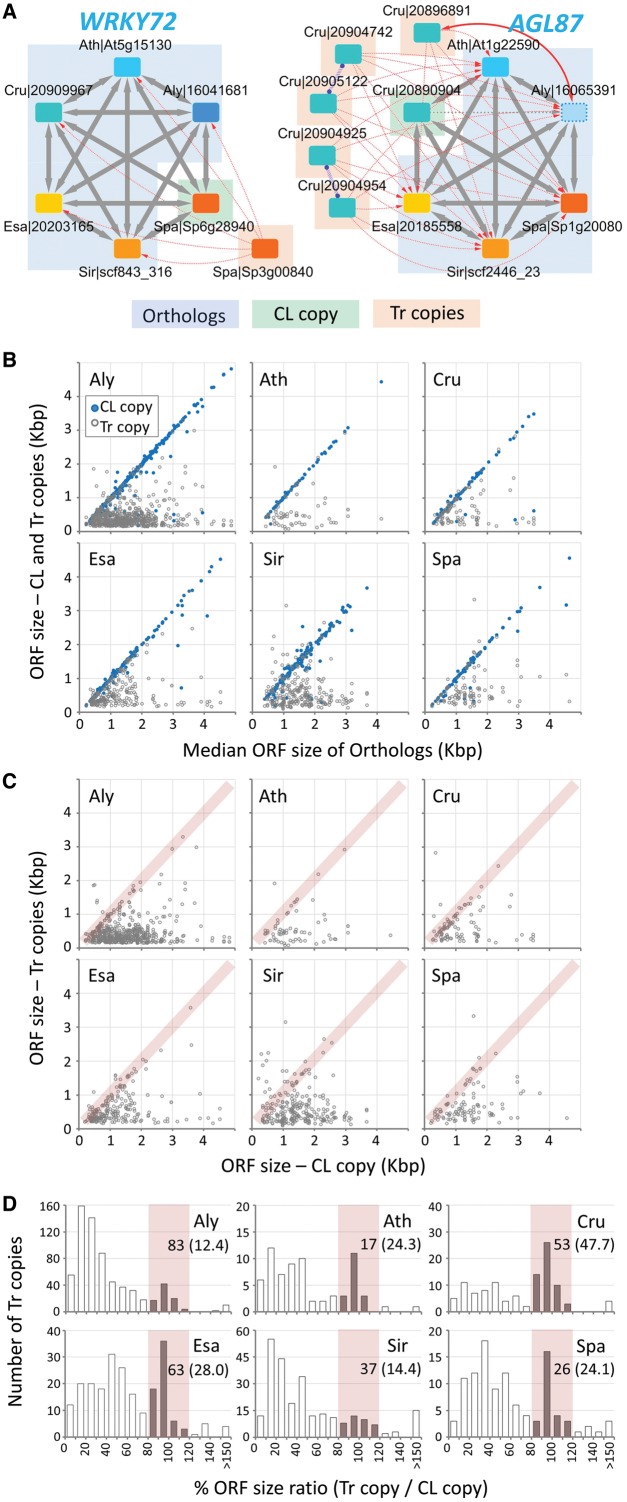
Characterization of lineage-specific transposition–duplication (*tr–d*) events among the six Brassicaceae genomes. (A) Examples of OrthNets with *tr–d* events specific for *S. parvula* (*WRKY DNA-BINDING PROTEIN 72*/*WRKY72*) and *C. rubella* (*AGAMOUS-LIKE 87*/*AGL87*). Within a *tr–d* event, the original donor copy (CL copy) is reciprocally co-linear to orthologues in other genomes (Orthologs), while transposed and duplicated paralogues (Tr copies) are not. Nodes and edges are as described in Fig. 3A. (B) Comparison between the ORF size of all loci involved in a *tr–d* event (i.e. both CL and Tr copies) with the median ORF size of Orthologs. Blue (filled) dots indicate CL copies. (C and D) ORF size comparison between Tr copies and their corresponding CL copy within each of the *tr–d* events, as a scatterplot (C) and a histogram of ORF size ratio (D). Pink shades indicate Tr copies with complete ORFs whose sizes are comparable (±20% in proportion) to that of the CL copy. Panel D also shows numbers and percentages (in parentheses) of Tr copies with complete ORFs below the species labels. The entire list of OrthNets showing lineage-specific *tr–d*, including CL and Tr copies, is in Supplementary Dataset S3.

**Figure 5.**
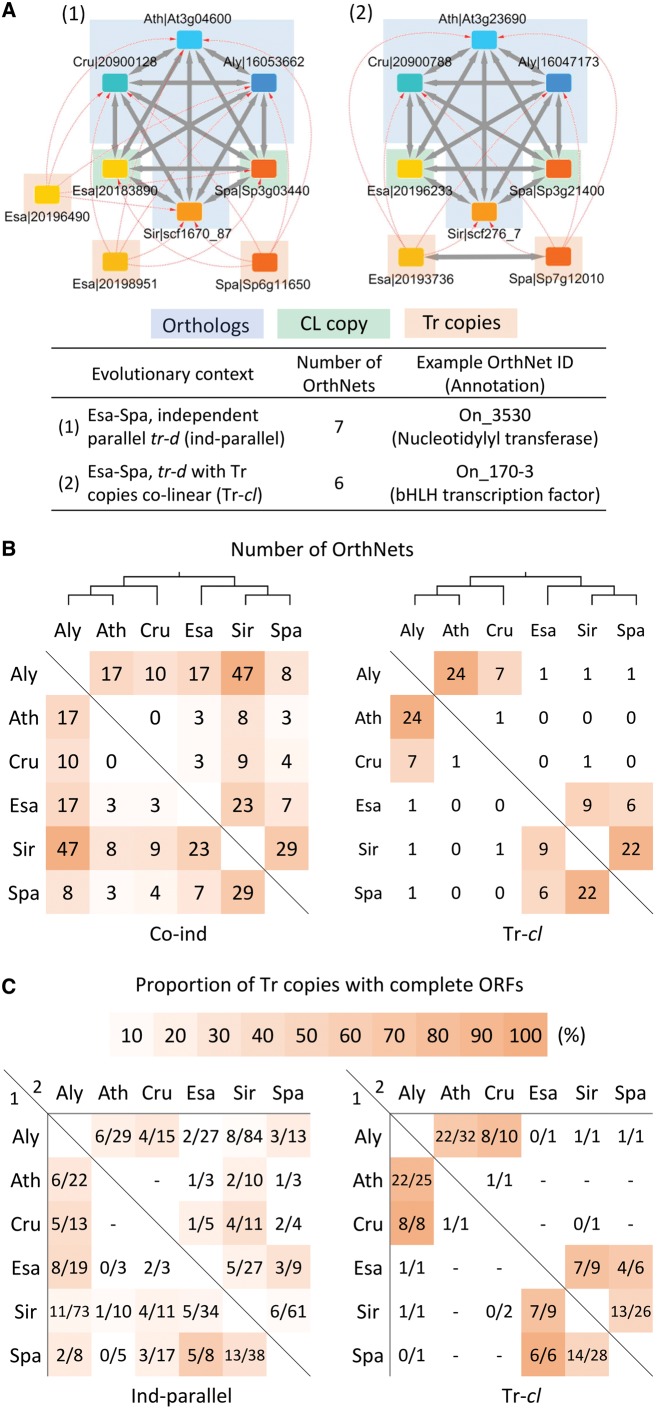
Transposition–duplication (*tr–d*) events shared by a pair of Brassicaceae genomes. (A) Example OrthNets with *tr–d* events shared by *E. salsugineum* and *S. parvula*, representing two categories: (i) independent-parallel (‘Ind-parallel’) *tr–d* events and (ii) *tr–d* events with Tr copies co-linear to each other (‘Tr–*cl*’). Nodes and edges are as described in Fig. 3A. (B) Number of OrthNets shared by pairs of genomes in ‘Ind-parallel’ and ‘Tr–*cl*’ categories, with the cladogram of the six crucifer genomes on the top. Heatmap colours visualize the rank of each cell based on the number of OrthNets in each category. (C) Proportion of Tr copies with complete ORFs (i.e. ORF size ±20% of the CL copy in proportion) within OrthNets presented in (B). The genome 1 (row)-genome 2 (column) position shows the number of Tr copies with complete ORF/total Tr copies in the genome 1, found in all OrthNets with *tr–d* shared by genomes 1 and 2. For example, the Aly–Ath and Ath–Aly positions in ‘Ind-parallel’ category indicate 6 out of 29 *A. lyrata* (Aly) Tr copies and 6 out of 22 *A. thaliana* (Ath) Tr copies, respectively, have complete ORFs in the 17 OrthNets with ‘Ind-parallel’ *tr–d* events shared by *A. lyrata* and *A. thaliana*. Heatmap colours indicate the percentage of Tr copies with complete ORFs, for cells with >5 total Tr copies.

We used the ‘search_OrthNet_pattern.py’ script in the ONfinder (Supplementary Fig. S2 and Table S3) to detect OrthNets representing *tr* and *tr–d* events either unique to each of the six Brassicaceae genomes or shared by more than one genome (Figs 4 and 5 and Supplementary Dataset S3). The number of OrthNets that showed *tr* events was smaller than those with *tr–d* events for all subsets of genomes including lineage-specific events (Supplementary Table S4). This observation agrees with the postulation that a *tr* event is a result of a deletion of the original/donor copy after a *tr–d* event.^1^

In OrthNets including *tr–d* events, we identified the original donor or co-linear (CL) copy, or copies if the donor locus included tandem duplications, and the acceptor or transposed (Tr) copies, based on properties of edges connecting each of the duplicated paralogues to its neighbouring nodes in other genomes (Figs 4A and 5A). Figure 4A represents OrthNets with *S. parvula* and *C. rubella* lineage-specific *tr–d* events for orthologues of the *WRKY72* and *AGL87*, respectively. The CL copy (Fig. 4A, ‘CL copy’) was a part of the hexagon and mostly reciprocally co-linear to its orthologue nodes (Fig. 4A, ‘Orthologs’) from other genomes. A Tr copy was connected to orthologue nodes in the hexagon through unidirectional *tr* edges (Fig. 4A, ‘Tr copies’). An OrthNet may contain a single lineage-specific *tr–d* event as in the OrthNet for *WRKY72* (Fig. 4A, left) or multiple events featuring one CL copy associated with multiple Tr copies. Also, Tr copies may further undergo tandem duplication as shown in the OrthNet for *AGL87* (Fig. 4A, right).

We compared the ORF sizes between CL and Tr copies with the median ORF size of the orthologues from other genomes in the hexagon for all OrthNets representing lineage-specific *tr–d* events. We observed a conservation of ORF sizes between most CL copies and their co-linear orthologues (Fig. 4B, blue/filled dots), while the majority of Tr copies had truncated ORFs (Fig. 4B, grey dots). We also found a small proportion of Tr copies which had ORFs that were of similar size to their respective CL copy (Fig. 4C and D, pink shades). The distribution of the ORF size ratio between Tr and the CL copy showed peaks at 80–120% (Fig. 4D, pink shades). These Tr copies that showed conservation in maintaining the original size of the ORFs were more abundant in *A. thaliana*, *C. rubella*, *E. salsugineum*, and *S. parvula*. These contributed to more than 24% of all Tr copies found in lineage-specific *tr–d* events in these genomes (Fig. 4D).

For *tr–d* events shared between any pair within the six genomes, we identified two categories with different evolutionary contexts: (i) parallel *tr–d* events independently occuring in two genomes (Fig. 5, ‘Ind-parallel’) and (ii) *tr–d* events where Tr copies from two genomes showing co-linearity between them (Fig. 5, ‘Tr–*cl*’). Figure 5A depicts examples of OrthNets including *tr–d* events in the two categories. We found a total of seven and six OrthNets with *tr–d* events in ‘Ind-parallel’ and ‘Tr–*cl*’ categories, respectively, shared between *E. salsugineum* and *S. parvula*. Genomes with higher TE and repetitive sequence contents, such as *A. lyrata*, *S. irio*, and, to a lesser extent, *E. salsugineum*, included more ‘ind-parallel’ *tr–d* events shared with other genomes (Fig. 5B, left panel). Among Tr copies in ‘ind-parallel’ *tr–d* events, the proportion of complete ORFs (i.e. ORF size within ±20% of the ORF of the corresponding CL copy) were comparable with Tr copies in LS *tr–d* events (Fig. 5C, left panel and Fig. 4D).

The ‘Tr–*cl*’ type *tr–d* events were mostly found between pairs of more recently diverged genomes, e.g. *A. lyrata*–*A. thaliana* and *S. irio*–*S. parvula*. The number of ‘Tr–*cl*’ *tr–d* events detected between Lineages I and II genomes were very low (Fig. 5B, right panel). This observation was consistent with the notion that such a rare event must involve a *tr–d* event before the divergence of the two Lineages followed by deletions in all species except for the two genomes compared. The proportion of Tr copies that retained complete ORFs compared with the CL copy in ‘Tr–*cl*’ type *tr–d* events was higher (≥50%) than that found for ‘ind-parallel’ type *tr–d* events (Fig. 5C).

### 3.5. Tr copies with complete ORFs were rare, but significantly more frequent than random chance

**Figure 6.**
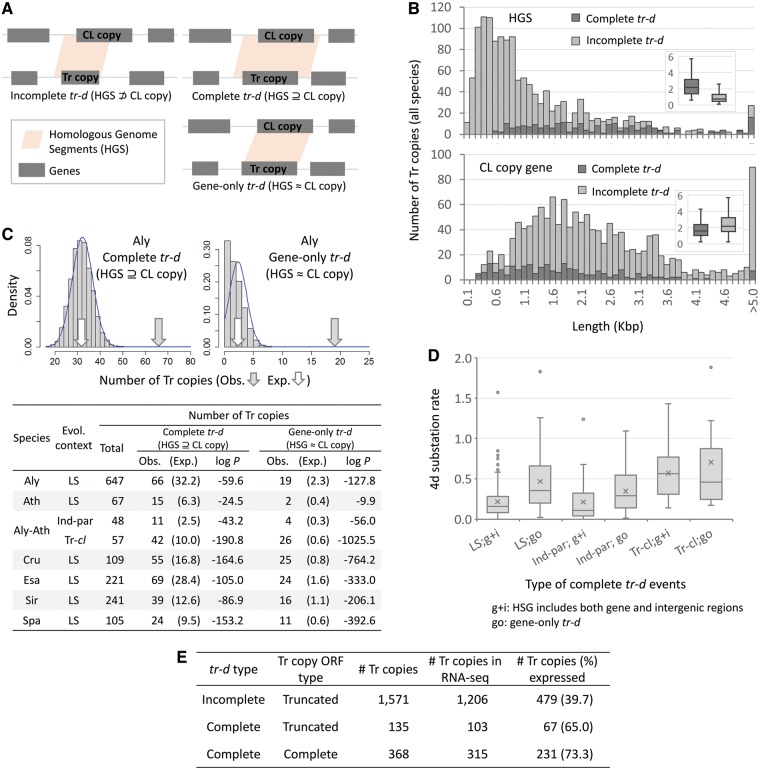
Characterization of duplicated genomic regions in transposition–duplication (*tr–d*) events. (A) We identified Homologous Genome Segments (HGSs) between the CL and Tr copy genes and adjacent genomic regions (±5 kb) in a *tr–d* event as described in Materials and Methods (2.5). A *tr–d* event is either complete or incomplete, based on whether the HGS included the full CL copy gene or not. A subset of complete *tr–d* events had HGS coinciding with the start and end of the CL copy gene without extending to the intergenic regions (‘gene-only’ *tr–d*). (B) Histograms and box-and-whisker plots (inlets) showing size distributions of HGSs and CL copy genes for complete and incomplete *tr–d* events. (C) Comparison of observed (Obs.) and expected (Exp.) occurrences of complete and gene-only *tr–d* events. Upper panel shows the distribution of expected occurrences from 10,000 random shuffling of HGSs and CL copy genes for *A. lyrata* (Aly)-specific complete and gene-only *tr–d* events. Fitting the random shuffling results to normal distributions (upper panel, blue curves) generated *P*-values of observed occurrences for *tr–d* events unique to each genome and the genus *Arabidopsis* (lower panel). (D) Four degenerate site (4d) substitution rates between ORFs of CL and Tr copy genes in different types of complete *tr–d* events. Complete *tr–d* events were either gene-only (‘go’) or with HGSs detected in both gene and intergenic regions (‘g+i’). We compared lineage-specific (LS) and shared *tr–d* events that are either independent-parallel (‘Ind-par’) or with Tr copies co-linear to each other (‘Tr–*cl*’). Lines and ‘x’ marks in the box indicate medians and means, respectively. (E) Proportion of Tr copy genes with expression evidences (RNA-seq FPKM>0) in all *tr–d* events either lineage-specific or shared by a pair of genomes. The *tr–d* type is as described in (A) and Tr copy ORF type is as in Fig. 4C and D (pink shade) and Fig. 6C. *S. irio* genes were excluded due to the lack of RNA-seq data.

We hypothesized that selection has favoured conservation of beneficial Tr copies to preserve the ORF in additional gene copies (Fig. 4C and D, pink shades; Fig. 5C), while majority of the Tr copies was either originally duplicated incompletely or have undergone mutations over time that had led to truncated ORFs. An alternative hypothesis is that these Tr copies with complete ORFs may have been easier to duplicate in their complete form by random chance due to their smaller gene size. Indeed, genes associated with Tr copies with complete ORFs were significantly shorter than those with Tr copies that had truncated ORFs (Fig. 6B and Supplementary Fig. S7).

To test our hypotheses, we shuffled duplicated genomic regions and duplicated genes in *tr–d* events. Then, we compared the occurrences of randomized *tr–d* events showing complete duplication of the entire CL copy gene with those observed among actual *tr–d* events (Fig. 6). First, to detect duplicated genomic regions in a *tr–d* event, we compared adjacent genomic regions, i.e. 5 kb up- and downstream regions, including the gene, of the CL copy and each of Tr copies. In this comparison, we searched for Homologous Genome Segments (HGSs) between the CL and Tr copy loci. As depicted in Fig. 6A, an incomplete *tr–d* event results in a HGS carrying only a part of the CL copy gene (HGS ⊅ CL copy), while in a complete *tr–d*, the HGS encompasses the entire CL copy gene (HGS ⊇ CL copy). Interestingly, we found a subset of complete *tr–d* events where the start and end positions of the HGS appeared to overlap with the start and end of the CL copy gene (HGS ≈ CL copy). We named this subset ‘gene-only’ *tr–d* (Fig. 6A) since the sequence homology was not detectable in the intergenic region further from the CL copy coding regions by more than 20% of the CL copy coding region size. A total of 224 complete *tr–d* events showed a shift towards longer HGSs, while their CL copy genes (coding regions) were significantly shorter (*P* < 0.001, two-tailed *t*-test), compared with those in the 1,166 incomplete *tr–d* events (Fig. 6B).

Following random shuffling of all HSGs and CL copy genes as described in Materials and Methods (2.5), we counted the occurrences of incomplete, complete, and gene-only *tr–d* events for each iteration. Figure 6C shows the comparison between the observed and expected occurrences of complete and gene-only *tr–d* events, where expected values were the mean values from 10,000 iterations. Assuming a normal distribution for the expected values, we estimated the *P*-value for the observed numbers of complete and gene-only LS *tr–d* events for each genome. Both complete and gene-only *tr–d* events were much more frequent than expected due to random chance. The gene-only *tr–d* events had smaller *P*-values than complete *tr–d* events in all categories tested except in *A. thaliana* lineage-specific *tr–d* events (Fig. 5C, table in the lower panel). We observe a smaller number of lineage-specific *tr–d* events in *A. thaliana* than in any other target genome. This may be a result of *A. thaliana* and *A. lyrata* being the closest among all pairs, included in the same genus. Hence, we included the *Arabidopsis* genus-specific *tr–d* events into consideration, which led to numbers and enrichment of complete and gene-only *tr-d* events comparable to other genomes (Fig. 5C, ‘Aly–Ath’).

Random occurrence of duplications cannot explain the observed proportion of complete *tr–d* events, which in >90% of the cases also resulted in complete ORFs in the Tr copy loci (e.g. Fig. 4C and D, pink shades). More likely, the observed proportion of complete and gene-only duplications was the sequential result of random duplications and selective retention of beneficial coding regions over time. This explanation is consistent with 4d substitution rates between complete ORFs of Tr copies and CL copies in *tr–d* events. Higher 4d substitution rates, as a proxy for older duplications, were found between ORFs of Tr and CL copy pairs in gene-only *tr–d* events (Fig. 6D, ‘go’). This was contrasting to *tr–d* events where HGSs comprised both gene and intergenic regions (Fig. 6D, ‘g + i’), for both lineage-specific (Fig. 6D, ‘LS’) and indepedent parallel (Fig. 6D, ‘Ind-par’) shared *tr–d* events. The 4d substitution rates associated with ‘Tr–*cl*’ type shared *tr–d* events (Fig. 6D, ‘Tr–*cl*’) showed median values comparable or higher than the median 4d substitution rates that represent the divergence between *A. thaliana* and Lineage II genomes (Supplementary Fig. S1D). This further agreed with the notion that a ‘Tr–*cl*’ type *tr–d* event was derived from duplications dated prior to the divergence of genomes that shared the events.

Complete *tr–d* events also included a higher number of Tr copy genes that showed evidence of expression compared with incomplete *tr–d* events (Fig. 6E). Incomplete *tr–d* was associated with most of the Tr copies with truncated ORFs, which comprised the majority of Tr copies in both lineage-specific *tr–d* (Fig. 4C and D) and independent parallel *tr–d* events shared by a pair of genomes (Fig. 5C). Out of total 1,706 Tr copies with truncated ORFs, only 135 were derived from complete *tr–d* events, in which the ORFs were most likely truncated by null mutations after the duplication (Fig. 6E). We found no enrichment of single exon genes, a signature of retrotransposons, among *tr–d* events (Supplementary Fig. S8).

### 3.6. Genes associated with lineage-specific and shared *tr–d* events

Supplementary Table S5 presents a partial list of OrthNets associated with lineage-specific *tr–d* events for each of the six Brassicaceae genomes, selected based on the most number of Tr copies with complete ORFs and expression evidences, except for *S. irio*, for which RNAseq data was not available. For each OrthNet listed in Supplementary Table S5, we included numbers of Tr copies tandem duplicated, with complete ORFs, and with expression evidences, as detailed in Supplementary Text S3. The complete list of OrthNets including lineage-specific *tr–d* events is available in Supplementary Dataset S3. We described genes and gene ontology terms enriched among them in lineage-specific *tr–d* events in Supplementary Text S3 and Dataset S4.

**Figure 7.**
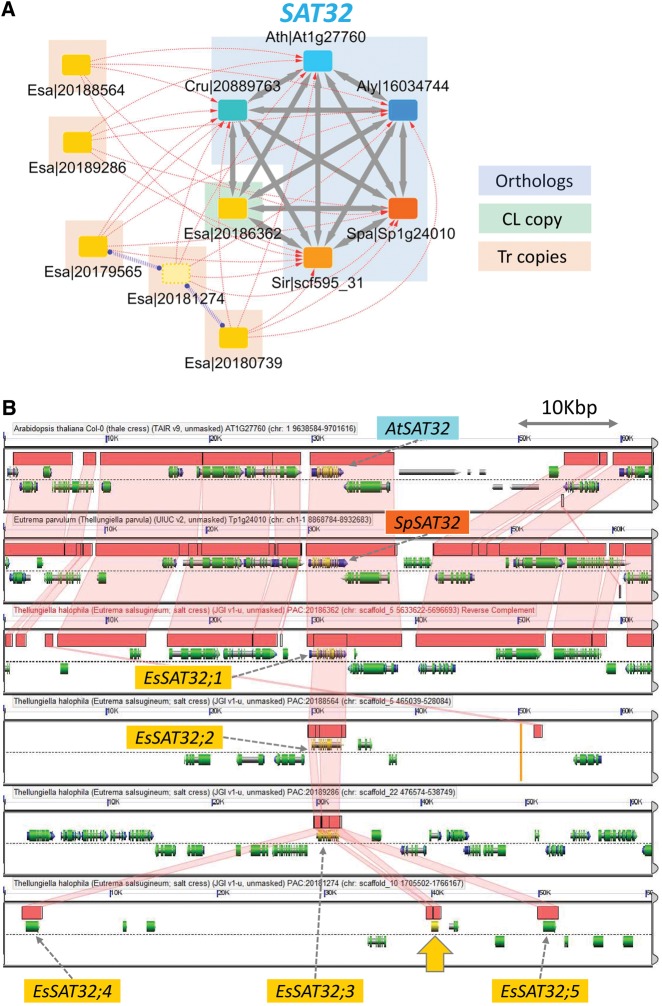
An exemplary OrthNet showing *E. salsugineum* lineage-specific *tr–d* events. (A) OrthNet for the *SALT-TOLERANCEE 32* (*SAT32*). Nodes and edges are as described in Fig. 3A. The OrthNet showed *E. salsugineum*-specific *tr–d*, with three of the five Tr copies tandem duplicated. (B) Comparison of *SAT32* loci and adjacent ±30 kb genomic regions between *A. thaliana*, *S. parvula*, and *E. salsugineum* as a GEvo plot (https://genomevolution.org/r/maxx (10 October 2018, date last accessed)). Pink/semi-transparent ribbons connect Homologous Genomic Segments (HGSs) between genomes, while gene models, mRNAs, and coding sequences are depicted as cylinders underneath HGSs (for detailed legends, see https://genomevolution.org/wiki/index.php/GEvo (10 October 2018, date last accessed)). *EsSAT32;1* is the CL copy (Esa|20186362), while *EsSAT32;2* (Esa|20188564), *EsSAT32;3* (Esa|20189286), *EsSAT32;4* (Esa|20179565), and *EsSAT32;5* (Esa|20180739) indicate the four Tr copies with complete ORFs. The yellow arrow marks the position of Esa|20181274, which contains a truncated ORF. *EsSAT32;3/4/5* showed intron losses without compromising gene products (see text and Supplementary Fig. S9 for details).

We selected the largest OrthNets with *E. salsugineum*-specific *tr–d* events (Supplementary Table S5) and independently visualized the extent of gene duplications using the GEvo tool in the CoGE database^35^ (Fig. 7). The *E. salsugineum* genome included six copies of *SALT TOLERANCE 32* (*SAT32*), which exists as a single copy in each of the other Brassicaceae genomes. Among five Tr copies detected for *EsSAT32*, we found three tandem duplicates (Fig. 7A, ‘Tr copies’). Four of the Tr copies had complete ORFs and three of them showed expression in either root or shoot tissues (Fig. 7A and Supplementary Table S5). The GEvo plot illustrates extensive sequence similarity among all loci and adjacent genomic regions that are reciprocally co-linear among them (Fig. 7B, *AtSAT32*, *SpSAT32*, and *EsSAT32;1*). Similar patterns were observed in comparisons with the *S. irio*, *C. rubella*, and *A. lyrata* co-linear orthologues (data not shown). The *EsSAT32* Tr copies (Fig. 7B, *EsSAT32;2/3/4/5*) represented examples of gene-only *tr–d* events (Fig. 6A), where sequence similarities were restricted to the expected border regions of the gene models (i.e. ±20% of the coding region size). Interestingly, the Tr copies *EsSAT32;3/4/5* also exhibited intron losses, resulting in 9, 1, and 1 exons, respectively, compared with the 13 exons in the CL copy *EsSAT32;1* (Fig. 6B), while maintaining high deduced amino acid similarities over most of the coding region (Supplementary Fig. S9). Among all *EsSAT* paralogues, the highest average expression was observed for one of the Tr copies, *EsSAT32;2* (Supplementary Dataset S1, OrthNet ID ON_2516 and gene ID 20188564).

For *tr–d* events shared by multiple genomes, we present the entire list of such OrthNets for all pairs of genomes, as well as those associated with Lineages I- and II-specific *tr–d* events, in Supplementary Dataset S3. The *tr–d* events shared by *E. salsugineum* and *S. parvula* were of particular interest, because they may indicate signatures of convergent evolution between these two species independently adapted to high salinity.^47^^,^^48^Supplementary Table S6 lists all OrthNets with ‘Ind-parallel’ and ‘Tr–*cl*’ type *tr–d* events (as defined in Fig. 5A) shared between these two extremophytes. We also included all Lineage II (i.e. *E. salsugineum*, *S. irio*, and *S. parvula*)-specific *tr–d* events that had truncated Tr copy ORFs only for *S. irio* (Supplementary Table S6, marked by superscript ‘e’). Among the ‘Ind-parallel’ *tr–d* events detected, three out of eight events were associated with stress signalling or response-related functions (Supplementary Table S6, *CDPK1*, *5PTASE11*, and *ABI1*). However, none of them included complete Tr copy ORFs in both *E. salsugineum* and *S. parvula*. Interestingly, the ‘Tr–*cl*’ category included more loci with Lineage II-specific *tr–d* followed by truncation of the Tr copy ORF in *S. irio*, leaving complete ORFs in the Tr copy loci only for *E. salsugineum* and *S. parvula*. Here, we found loci encoding orthologues of a putative basic helix-loop-helix (bHLH) type transcription factor, a NAC transcription factor (NAC058), and a calcineurin B-like protein 10 (CBL10). All Tr copies encoding these regulatory proteins showed expression evidence in both halophytes (Supplementary Table S6).

## 4. Discussion

### 4.1. A systematic identification of orthologous loci with the same evolutionary history

While a number of tools are available to detect co-linearity or synteny blocks among multiple genomes,^32^^,^^33^^,^^49^^,^^57^^,^^58^ there has been a lack of methods which can systematically identify all groups of orthologous gene loci among multiple genomes that underwent the same set of evolutionary events, such as gene duplications and transpositions in a certain lineage or multiple lineages that are either mono-, para-, or polyphyletic. In an attempt to fill this gap, we developed the CLfinder-OrthNet pipeline. The CLfinder module detects co-linearity and transposition for individual loci, rather than for synteny blocks, to facilitate identification of single-gene transposition that consist the majority of transposition events^1^^,^^22^ and automatically generates a summary of all pairwise genome comparisons as exemplified in Table 1. The ONfinder module follows the CLfinder and produces OrthNets connecting orthologous genes with edges representing the presence or absence of co-linearity among them, enabling a search based on the network topology for all orthologous gene groups sharing an evolutionary history.

Previous works have suggested network representation of synteny among orthologues as an effective method to combine and summarize synteny blocks identified by all-to-all pairwise comparisons among multiple genomes.^59^ Synteny networks connecting co-linear orthologues from multiple genomes with undirected edges traced the evolutionary path of a gene family.^25^ This approach has been used to compare the extent of gene duplications and lineage-specific expansion of gene families between mammalian and plant genomes.^26^ While the CLfinder module similarly performs all-to-all pairwise analyses to detect co-linearity in gene order, OrthNets detected by the CLfinder-OrthNet pipeline are different from synteny networks^59^ in a number of ways. For example, while synteny networks connected co-linear nodes with undirected edges, OrthNets connected nodes with directional edges with co-linearity or lack of it (i.e. transposed) encoded as edge properties. An OrthNet includes orthologues connected by reciprocal edges as well as paralogues derived from duplications connected by unidirectional edges to their neighbouring nodes found in other genomes [Fig. 3A, panels (4) and (5); Figs 4A and 5A]. We aimed to separate each OrthNet into a unit that represents a group of orthologues and paralogues likely derived from a single ancestral locus, by employing Markov clustering (MCL) (Supplementary Text S2). We chose MCL to control edge weights to prefer undirected tandem duplicated edges and reciprocal edges over unidirectional edges during the clustering process. In this way, each of the majority of OrthNets, e.g. >85% of all OrthNets in case of the six Brassicaceae genomes (Fig. 3B), represents the evolutionary history of genes derived from a single ancestral locus as the network topology. Essentially, OrthNets enable detection of all loci from multiple genomes that share the same evolutionary history by a search using a given network topology as the query (e.g. Figs 3A and 5A). We used this functionality to characterized transposition–duplication (*tr–d*) events in six Brassicaceae genomes, as a proof-of-concept.

### 4.2. Transposition–duplication as a major mechanism for erosion of co-linearity

For the transposition and transposition–duplication (*tr–d*) of non-TE gene loci, two types of models, retrotransposon-associated and DNA repair or replication-associated models have been suggested as the main mechanisms.^60^^,^^61^ A *tr–d* event derived from retrotransposons often leads to duplication of single exon genes.^62–64^ Transposition–duplication may also arise during the non-homologous end-joining (NHEJ) repair process of DNA double-strand breaks (DSB), where a short sequence motif may act as an anchor to a foreign sequence to fill-in a gap.^1^ In agreement with this model, a previous comparison of *A. lyrata* and *A. thaliana* found a significant enrichment of flanking repeats, as short as 15 bps, among transposed genes.^65^ The correlation between the proportion of query gene loci showing distal displacement (Supplementary Fig. S1C, *d_n_*_,_*_n+_*_1_>20 or ‘Diff Chr’) and overall TE contents of the query genome, rather than divergence time (Supplementary Fig. S1D), supports the DSB-repair model. Higher TE contents likely provide a higher frequency of short repeat anchors required for the NHEJ DSB-repair, and TE activities themselves may also cause the DSB that lead to such repairs.^1^ The DSB-repair model can explain *tr–d* of multi-exon genes, which constitute the majority of *tr–d* events found in this study (Supplementary Fig. S8).

Among lineage-specific *tr–d* events captured in OrthNets, we found a subset of transposed–duplicated gene loci (Fig. 4A, Tr copies) retaining similar ORF sizes compared with their respective donor locus (Fig. 4A, CL copy), as well as to orthologues in other species (Fig. 4B–D). The DSB repair model of *tr–d* suggests that the duplicated region may start and end virtually at any random position in a genome, given that the short sequence motif needed for the repair is likely ubiquitously available and can be as short as several nucleotides.^1^^,^^65^ However, our simulation reveled that both ‘complete’ and ‘gene-only’ *tr–d* events were far more frequent than what was expected from a random duplication model alone (Fig. 6C). We are not aware of a gene duplication mechanism that preferably duplicates non-TE, protein-coding, multi-exon genes as entire units. Rather, our observation common to all six tested crucifer genomes is likely a result of random *tr–d* events (e.g. through DSB repair), followed by accumulation of mutations throughout the duplicated regions, except where the complete coding sequences were selectively retained. Supporting this notion, Tr copy genes with complete ORFs were more frequent among shared *tr–d* of older ‘Tr–*cl*’ type events (Figs 5C and 6D). These were also more likely to be expressed, hence less likely to be pseudogenes, compared with Tr copies with truncated ORFs (Fig. 6E). See Supplementary Texts for further discussions on the age of ‘gene-only’ *tr–d* events (Supplementary Text S4) and on *tr–d* frequencies and TE contents (Supplementary Text S5). Overall, our analyses depicted the landscape of *tr–d* events among Brassicaceae genomes, where the majority of *tr–d* was incomplete, while small numbers of *tr–d* including complete Tr copy ORFs and gene-only *tr–d* were likely to have resulted from random duplication events followed by selective retention of coding sequences over time.

### 4.3. Search for extremophyte-specific *tr–d* events using CLfinder-OrthNet

One possible application of the CLfinder-OrthNet pipeline is to retrieve orthologues sharing evolutionary events unique to a lineage with a specific trait or multiple lineages exhibiting a convergent trait, e.g. the two extremophyte *S. parvula* and *E. salsugineum*. As detailed in Supplementary Text S6, these two genomes have been identified with gene copy number and structural variations compared with *A. thaliana* that were associated with stress-adapted traits.^10^^,^^66^^,^^67^ In this study, CLfinder-OrthNet identified 63, 26, and 14 orthologue groups, represented as OrthNets, showing gene copy number increases through *tr–d* events specific to *E. salsugineum*, *S. parvula*, and both, respectively (Fig. 4D, Supplementary Table S6, and Dataset S3). These numbers are orders of magnitude fewer than previous searches from a pairwise comparison with *A. thaliana*,^5^ signifying the vastly improved resolution in finding extremophyte-specific events.

The OrthNet for the *SALT-TOLERANCE 32* (*SAT32*) locus (Fig. 7A and Supplementary Table S5, ON_2516) represents the largest *E. salsugineum*-specific *tr–d* event. *SAT32* encodes a transcription regulator, whose expression level positively correlated with the survival rate of the model plant *A. thaliana* under salt stress.^68^ Three of the four *EsSAT32* paralogues with complete ORFs exhibited intron losses (Fig. 7B andSupplementary Fig. S9). Intron losses and smaller transcript sizes are reported to enable regulation of expression timing in Drosophila and mouse.^69^^,^^70^ It is not clear whether ‘gene-only’ *tr–d* events (Fig. 7B) among *EsSAT32* paralogues is indicative of reverse transcriptase-mediated duplication leading to intron losses^71^ or different rate of mutation between gene and intergenic regions. Either way, such variation in intergenic regions including promoter regions may lead to sub-functionalization.^2^ At least three *EsSAT32* paralogues exhibited different basal expression strengths in root and shoot tissues (data not shown).

A notable example of *S. parvula-*specific *tr–d*, with copy number increases in complete ORFs, is the *ZRT/IRT-LIKE PROTEIN 3* (*ZIP3*) locus encoding a zinc transporter (Supplementary Table S5). This particular *tr–d* may be a signature of an adaptation in *S. parvula*, to soils that are highly saline and also depleted in micronutrients such as zinc and iron in central Anatolia.^72^^,^^73^ See Supplementary Text S7 and S8 for discussions on genes involved in *tr–d* unique to each extremophyte, as well as *tr–d* shared by the two extremophytes.

### 4.4. Concluding remarks: CLfinder-OrthNet, a flexible toolkit for comparative genomics

The CLfinder-OrthNet pipeline, in a proof-of-concept application, successfully encodes more than 85% of entire loci among six Brassicaceae genomes into OrthNet units in which evolutionary histories of genes derived from single ancestral loci can be traced (Fig. 3B). Using a network topology-based search, we identified groups of orthologues, represented as OrthNets that share the same evolutionary histories (Fig. 3A), including *tr–d* unique to any subset of the six Brassicaceae genomes (Figs 4 and 5, Supplementary Dataset S3).

As detailed in Supplementary Text S9, CLfinder-OrthNet offers multiple options to apply the pipeline flexibly depending on target genomes and goals of the study. The sensitivity and stringency of co-linearity detection are adjustable by controlling parameters depending on the range of target genomes. The CLfinder module can use results from any method of sequence clustering and comparison, as well as genomic features other than protein-coding genes, as inputs. Moreover, the two modules can be used separately. For example, researchers can use the CLfinder module to quickly summarize the distribution of co-linear, tandem duplicated, and transposed genes among multiple genomes (e.g. Table 1), while the ONfinder module can accept locus-level synteny information from other methods to generate OrthNets.

Overall, the CLfinder-OrthNet pipeline offers a flexible toolkit to compare the arrangement of gene and other genomic features among multiple genomes. Future applications include, but not limited to, tracing evolutionary histories of a gene or gene families; inference of orthology based on both sequence homology and co-linearity; studying incongruence between sequence homology and synteny; and identification of candidate gene copy number variations associated with specific hypothesis-driven evolutionary mechanisms or traits.

## Funding

This work was supported by National Science Foundation (MCB 1616827) and the Next Generation BioGreen21 Program (PJ01317301) of the Rural Development Administration, Republic of Korea.

## Supplementary data

Supplementary data (Supplementary Texts S1-S9, Tables S1-S6, Figures S1-S9, and Dataset S2-S4) are available at *DNARES* online. Supplementary Dataset S1 is deposited at figshare (https://doi.org/10.6084/m9.figshare.6959435.v1). The CLfinder-OrthNet pipeline is available in a GitHub (https://github.com/ohdongha/CL_finder).

## Conflict of interest

None declared. 

## Supplementary Material

Supplementary Data
