# Supplementary material for: Landscape of gene transposition–duplication within the Brassicaceae family
Source: DNA Res. 2018 Oct 30;26(1):21–36. doi: 10.1093/dnares/dsy035 (PMC6379040; doi:10.1093/dnares/dsy035)
Supplement: Supplementary Data [file dsy035_supp.zip › dsy035-suppl_data/Oh_Dassanayake_Supplementary_Table_Figures_re2-revised.pdf]

Landscape of gene transposition-duplication within the Brassicaceae family

Dong-Ha Oh and Maheshi Dassanayake

Department of Biological Sciences, Louisiana State University, Baton Rouge, LA 70803

Correspondence: Dong-Ha Oh (ohdongha@gmail.com), Maheshi Dassanayake (maheshid@lsu.edu)

SUPPLEMENTARY TABLEs, FIGURES, AND DATASETS

Supplementary Tables and Figures:

Table S1. Gene model resources used for the construction of the Brassicaceae phylogenetic tree in Figure 1.

Table S2. Expected number of co-linear loci between randomly shuffled genomes

Table S3. Regular expressions to retrieve OrthNets containing transposition (*tr*) and transposition-dupcliation (*tr-d*) events unique to a genome and shared by a pair of genomes or within Lineage I and II, among the six Brassicaceae genomes.

Table S4. Number of OrthNets showing lineage-specific (LS) transposition (*tr*) and transposition-duplication (*tr-d*)

Table S5. A partial list of OrthNets associated with lineage-specific (LS) transposition-duplication (*tr-d*) events

Table S6. List of OrthNets including *tr-d* events shared by *E. salsugineum* (Esa) and *S. parvula* (Spa)

Figure S1. Erosion of co-linearity between Brassicaceae (crucifer family) genomes`

Figure S2. A detailed flowchart of the CLfinder-OrthNet pipeline.

Figure S3. The CLfinder process explained with an example.

Figure S4. An example of Markov clustering (MCL) process to resolve OrthNets containing outparalogs

Figure S5. An example of OrthNet including Aly-specific *tr-d* events with a large number of duplicated copies

Figure S6. Comparison of orthologous gene clusters identified by OrthNet (ON) and OrthoFinder (OF).

Figure S7. Comparison of CL copy gene lengths between Tr copies with complete (-c) and truncated (-t) ORFs in six Brassicaceae genomes

Figure S8. Proportion of single-exon genes among CL and Tr copies in lineage-specific (LS) and shared *tr-d* events, compared to all genes in six Brassicaceae genomes.

Figure S9. Alignment of deduced amino acid sequences for Brassicaceae *SALT TOLERANCE 32* (*SAT32*) genes.

Supplementary dataset files available as separate files:

Dataset S1. Combined output of the CLfinder-OrthNet analysis for the six Brassicaceae genomes, with RNAseq expression evidence information (deposited at: https://doi.org/10.6084/m9.figshare.6959435.v1)

Dataset S2. All OrthNets derived from the analysis of the six Brassicaceae genomes, formatted as Simple Interaction Files (SIF).

Dataset S3. List of OrthNets that include transposition and transposition-duplication events unique to a genome or shared by groups of genomes among the six Brassicaceae genomes.

Dataset S4. List of Gene Ontology (GO) terms enriched in genes associated with LS *tr-d* events

Table S1. Gene model resources used for the construction of the Brassicaceae phylogenetic tree in Figure 1.

Species (NCBI Taxonomy ID)	Source[a]	Version[b]	URL
Aethionema arabicum (228871)	CoGE	19578	https://genomevolution.org/coge/GenomeInfo.pl?gid=19578
Brassica napus (3708)	BRAD	5	http://brassicadb.org/brad/datasets/pub/Genomes/Brassica_napus
Brassica oleracea (3712)	BRAD	1.1	http://brassicadb.org/brad/datasets/pub/Genomes/Brassica_oleracea/V1.1
Brassica rapa (3711)	BRAD	1.5	http://brassicadb.org/brad/datasets/pub/Genomes/Brassica_rapa/V1.0/V1.5
Camelina sativa (90675)	BRAD	2	http://brassicadb.org/brad/datasets/pub/BrassicaceaeGenome/Camelina_sativa
Capsella grandiflora (264402)	CoGE	25807	https://genomevolution.org/coge/GenomeInfo.pl?gid=25807
Leavenworthia alabamica (310722)	CoGE	19577	https://genomevolution.org/coge/GenomeInfo.pl?gid=19577
Raphanus raphanistrum (109996)	CoGE	25862	https://genomevolution.org/coge/GenomeInfo.pl?gid=22862
Raphanus sativus (3726)	CoGE	25866	https://genomevolution.org/coge/GenomeInfo.pl?gid=22866
Tarenaya hassleriana (28532)	CoGE	22586	https://genomevolution.org/coge/GenomeInfo.pl?gid=22586

[a] CoGE: https://genomevolution.org; BRAD: http://brassicadb.org [b] Genome ID for resources from CoGE

Table S2. Expected number of co-linear loci between randomly shuffled genomes

CLfinder parameters [a]			Co-linear loci between simulated genomes with 27,000 loci [b]	
W	*N*	*G*	Number	Proportion (%)
20	2	20	1479.08 ± 52.28	5.48 ± 0.19
**20**	**3**	**20**	**39.43 ± 9.85**	**0.15 ± 0.04**
20	4	20	0.63 ± 1.29	0.00 ± 0.00

[a] The window size (*W*), the number of co-linear loci-in-chain threshold (*N*), and the maximum gap allowed between co-linear loci-in chain (*G*), as in Figure S2. The bold-faced parameters were used in the analysis of the six target Brassicaceae genomes.

[b] Mean ± standard deviation of values from 10,000 simulations by randomly shuffling gene loci.

Table S3. Query patterns to retrieve OrthNets containing transposition (*tr*) and transposition-duplication (*tr-d*) events unique to a genome and shared by a subset among the six Brassicaceae genomes.

Type [a]	Shared by	Node copy number query [b]						Search target	Edge-type pattern query [c]
		Aly	Ath	Cru	Esa	Sir	Spa		
Lineage-specific *tr-d*	Aly	>1	1	1	1	1	1	Aly	tr.tr.tr.tr.tr
	Ath	1	>1	1	1	1	1	Ath	tr.tr.tr.tr.tr
	Cru	1	1	>1	1	1	1	Cru	tr.tr.tr.tr.tr
	Esa	1	1	1	>1	1	1	Esa	tr.tr.tr.tr.tr
	Sir	1	1	1	1	>1	1	Sir	tr.tr.tr.tr.tr
	Spa	1	1	1	1	1	>1	Spa	tr.tr.tr.tr.tr
Shared *tr-d* occurred independently in parallel (ind-par)	Aly-Ath	>1	>1	1	1	1	1	Aly	(tr|nd).(tr|nd).(tr|nd).(tr|nd).(tr|nd)
		>1	>1	1	1	1	1	Ath	(tr|nd).(tr|nd).(tr|nd).(tr|nd).(tr|nd)
	Aly-Cru	>1	1	>1	1	1	1	Aly	(tr|nd).(tr|nd).(tr|nd).(tr|nd).(tr|nd)
		>1	1	>1	1	1	1	Cru	(tr|nd).(tr|nd).(tr|nd).(tr|nd).(tr|nd)
	Ath-Cru	1	>1	>1	1	1	1	Ath	(tr|nd).(tr|nd).(tr|nd).(tr|nd).(tr|nd)
		1	>1	>1	1	1	1	Cru	(tr|nd).(tr|nd).(tr|nd).(tr|nd).(tr|nd)
	Esa-Sir	1	1	1	>1	>1	1	Esa	(tr|nd).(tr|nd).(tr|nd).(tr|nd).(tr|nd)
		1	1	1	>1	>1	1	Sir	(tr|nd).(tr|nd).(tr|nd).(tr|nd).(tr|nd)
	Esa-Spa	1	1	1	>1	1	>1	Esa	(tr|nd).(tr|nd).(tr|nd).(tr|nd).(tr|nd)
		1	1	1	>1	1	>1	Spa	(tr|nd).(tr|nd).(tr|nd).(tr|nd).(tr|nd)
	Sir-Spa	1	1	1	1	>1	>1	Sir	(tr|nd).(tr|nd).(tr|nd).(tr|nd).(tr|nd)
		1	1	1	1	>1	>1	Spa	(tr|nd).(tr|nd).(tr|nd).(tr|nd).(tr|nd)
	Aly-Esa	>1	1	1	>1	1	1	Aly	(tr|nd).(tr|nd).(tr|nd).(tr|nd).(tr|nd)
		>1	1	1	>1	1	1	Esa	(tr|nd).(tr|nd).(tr|nd).(tr|nd).(tr|nd)
	Aly-Sir	>1	1	1	1	>1	1	Aly	(tr|nd).(tr|nd).(tr|nd).(tr|nd).(tr|nd)
		>1	1	1	1	>1	1	Sir	(tr|nd).(tr|nd).(tr|nd).(tr|nd).(tr|nd)
	Aly-Spa	>1	1	1	1	1	>1	Aly	(tr|nd).(tr|nd).(tr|nd).(tr|nd).(tr|nd)
		>1	1	1	1	1	>1	Spa	(tr|nd).(tr|nd).(tr|nd).(tr|nd).(tr|nd)
	Ath-Esa	1	>1	1	>1	1	1	Ath	(tr|nd).(tr|nd).(tr|nd).(tr|nd).(tr|nd)
		1	>1	1	>1	1	1	Esa	(tr|nd).(tr|nd).(tr|nd).(tr|nd).(tr|nd)
	Ath-Sir	1	>1	1	1	>1	1	Ath	(tr|nd).(tr|nd).(tr|nd).(tr|nd).(tr|nd)
		1	>1	1	1	>1	1	Sir	(tr|nd).(tr|nd).(tr|nd).(tr|nd).(tr|nd)
	Ath-Spa	1	>1	1	1	1	>1	Ath	(tr|nd).(tr|nd).(tr|nd).(tr|nd).(tr|nd)
		1	>1	1	1	1	>1	Spa	(tr|nd).(tr|nd).(tr|nd).(tr|nd).(tr|nd)
	Cru-Esa	1	1	>1	>1	1	1	Cru	(tr|nd).(tr|nd).(tr|nd).(tr|nd).(tr|nd)
		1	1	>1	>1	1	1	Esa	(tr|nd).(tr|nd).(tr|nd).(tr|nd).(tr|nd)
	Cru-Sir	1	1	>1	1	>1	1	Cru	(tr|nd).(tr|nd).(tr|nd).(tr|nd).(tr|nd)
		1	1	>1	1	>1	1	Sir	(tr|nd).(tr|nd).(tr|nd).(tr|nd).(tr|nd)
	Cru-Spa	1	1	>1	1	1	>1	Cru	(tr|nd).(tr|nd).(tr|nd).(tr|nd).(tr|nd)
		1	1	>1	1	1	>1	Spa	(tr|nd).(tr|nd).(tr|nd).(tr|nd).(tr|nd)

(This table is continued to the next page)

Table S3. (continued from the previous page)

Type [a]	Shared by	Node copy number query [b]						Search target	Edge-type pattern query [c]
		Aly	Ath	Cru	Esa	Sir	Spa		
Shared *tr-d* with Tr copies co-linear to each other (Tr-*cl*)	Aly-Ath	>1	>1	1	1	1	1	Aly	cl.(tr|nd).(tr|nd).(tr|nd).(tr|nd)
		>1	>1	1	1	1	1	Ath	cl.(tr|nd).(tr|nd).(tr|nd).(tr|nd)
	Aly-Cru	>1	1	>1	1	1	1	Aly	(tr|nd).cl.(tr|nd).(tr|nd).(tr|nd)
		>1	1	>1	1	1	1	Cru	cl.(tr|nd).(tr|nd).(tr|nd).(tr|nd)
	Ath-Cru	1	>1	>1	1	1	1	Ath	(tr|nd).cl.(tr|nd).(tr|nd).(tr|nd)
		1	>1	>1	1	1	1	Cru	(tr|nd).cl.(tr|nd).(tr|nd).(tr|nd)
	Esa-Sir	1	1	1	>1	>1	1	Esa	(tr|nd).(tr|nd).(tr|nd).cl.(tr|nd)
		1	1	1	>1	>1	1	Sir	(tr|nd).(tr|nd).(tr|nd).cl.(tr|nd)
	Esa-Spa	1	1	1	>1	1	>1	Esa	(tr|nd).(tr|nd).(tr|nd).(tr|nd).cl
		1	1	1	>1	1	>1	**Spa**	**(tr|nd).(tr|nd).(tr|nd).cl.(tr|nd)**
	Sir-Spa	1	1	1	1	>1	>1	Sir	(tr|nd).(tr|nd).(tr|nd).(tr|nd).cl
		1	1	1	1	>1	>1	Spa	(tr|nd).(tr|nd).(tr|nd).(tr|nd).cl
	Aly-Esa	>1	1	1	>1	1	1	Aly	(tr|nd).(tr|nd).cl.(tr|nd).(tr|nd)
		>1	1	1	>1	1	1	Esa	cl.(tr|nd).(tr|nd).(tr|nd).(tr|nd)
	Aly-Sir	>1	1	1	1	>1	1	Aly	(tr|nd).(tr|nd).(tr|nd).cl.(tr|nd)
		>1	1	1	1	>1	1	Sir	cl.(tr|nd).(tr|nd).(tr|nd).(tr|nd)
	Aly-Spa	>1	1	1	1	1	>1	Aly	(tr|nd).(tr|nd).(tr|nd).(tr|nd).cl
		>1	1	1	1	1	>1	Spa	cl.(tr|nd).(tr|nd).(tr|nd).(tr|nd)
	Ath-Esa	1	>1	1	>1	1	1	Ath	(tr|nd).(tr|nd).cl.(tr|nd).(tr|nd)
		1	>1	1	>1	1	1	Esa	(tr|nd).cl.(tr|nd).(tr|nd).(tr|nd)
	Ath-Sir	1	>1	1	1	>1	1	Ath	(tr|nd).(tr|nd).(tr|nd).cl.(tr|nd)
		1	>1	1	1	>1	1	Sir	(tr|nd).cl.(tr|nd).(tr|nd).(tr|nd)
	Ath-Spa	1	>1	1	1	1	>1	Ath	(tr|nd).(tr|nd).(tr|nd).(tr|nd).cl
		1	>1	1	1	1	>1	Spa	(tr|nd).cl.(tr|nd).(tr|nd).(tr|nd)
	Cru-Esa	1	1	>1	>1	1	1	Cru	(tr|nd).(tr|nd).cl.(tr|nd).(tr|nd)
		1	1	>1	>1	1	1	Esa	(tr|nd).(tr|nd).cl.(tr|nd).(tr|nd)
	Cru-Sir	1	1	>1	1	>1	1	Cru	(tr|nd).(tr|nd).(tr|nd).cl.(tr|nd)
		1	1	>1	1	>1	1	Sir	(tr|nd).(tr|nd).cl.(tr|nd).(tr|nd)
	Cru-Spa	1	1	>1	1	1	>1	Cru	(tr|nd).(tr|nd).(tr|nd).(tr|nd).cl
		1	1	>1	1	1	>1	Spa	(tr|nd).(tr|nd).cl.(tr|nd).(tr|nd)
tr-d shared within either Lineage I or II	Lineage I	>1	>1	>1	1	1	1	Aly	(cl|nd).(cl|nd).(tr|nd).(tr|nd).(tr|nd)
		>1	>1	>1	1	1	1	Ath	(cl|nd).(cl|nd).(tr|nd).(tr|nd).(tr|nd)
		>1	>1	>1	1	1	1	Cru	(cl|nd).(cl|nd).(tr|nd).(tr|nd).(tr|nd)
	Lineage II	1	1	1	>1	>1	>1	Esa	(tr|nd).(tr|nd).(tr|nd).(cl|nd).(cl|nd)
		1	1	1	>1	>1	>1	Sir	(tr|nd).(tr|nd).(tr|nd).(cl|nd).(cl|nd)
		1	1	1	>1	>1	>1	Spa	(tr|nd).(tr|nd).(tr|nd).(cl|nd).(cl|nd)

[a] The type of shared *tr-d* events are as described in Figure 5A of the main manuscript. OrthNets including *tr-d* events were identified using two criteria: (1) node copy numbers in each genome [b] and (2) presence of a node from the "search target" species that matches the "edge-type pattern" query expressed as a python regular expression [c]. The edge-type pattern specifies the type of edges that connect the node to its neighbors from each species, in the order of Aly, Ath, Cru, Esa, Sir, and Spa, except for the search target species itself, separated by periods. Python regular expression (tr|nd) and (cl|nd) mean either tr or nd and cl or nd types are allowed. For example, if search target is "Spa" and edge-type pattern query is "(tr|nd).(tr|nd).(tr|nd).cl.(tr|nd)," this query (bold-faced in the table) looks for a Spa node that are connected to nodes from all species except for Esa with either *tr* or *nd* edges, while connected to an Esa node by a *cl* edge. We selected *tr-d* events shared by multiple genomes only when queries from each of all search targets finds a node matching the edge-type pattern query.

Table S4. Number of OrthNets showing lineage-specific (LS) transposition (*tr*) and transposition-duplication (*tr-d*)

	Aly	Ath	Cru	Esa	Sir	Spa
LS *tr*	12	5	25	38	30	9
LS *tr-d*	421	64	85	175	172	86
TE (%)	30.7	18.2	19.3	48.8	32.7	16.7

Example OrthNets representing LS *tr* and LS *tr-d* events are in Fig. 4A panel (3) and (5), respectively. Transposable element (TE) contents were obtained as described in Methods.

Table S5. A partial list of OrthNets associated with lineage-specific (LS) transposition-duplication (*tr-d*) events [a]

LS *tr-d*	OrthNet ID (ON_)	Node count pattern [b]	Annotation	CL copy [c] ORF length (nt) / # exons	Number of Tr copies: Total (*td*), cORF, exp, cORF & exp [d]	Number of Tr copies: Duplication mode: g+i, go [e]
Aly	1227	11.1.1.1.1.1	unknown protein	630 / 5	5 (3), 4, 2, 2	5, 0
	3580	4.1.1.1.1.1	cytochrome c oxidase 15 (COX15)	1374 / 7	2, 2, 2, 2	2, 0
	4486	3.1.1.1.1.1	carbamoyl phosphate synthetase A (CARA)	1293 / 10	2, 2, 2, 2	0, 2
	3527	4.1.1.1.1.1	RNA-dependent RNA polymerase 1 (RDR1)	3324 / 3	3, 1, 1, 1	1, 0
	3557	4.1.1.1.1.1	beta-amylase 3 (BMY3)	1605 / 3	3, 1, 2, 1	1, 0
	4003	3.1.1.1.1.1	peroxin4 (PEX4)	474 / 5	1, 1, 1, 1	0, 1
	200-3	3.1.1.1.1.1	homeobox protein 5 (HB5)	942 / 3	2, 1, 2, 1	1, 0
Ath	4411	1.3.1.1.1.1	PAI3*	828 / 5	2, 2, 2, 2	1, 1
	4622	1.2.1.1.1.1	HPA1*	1254 / 7	1, 1, 1, 1	1, 0
	5135	1.2.1.1.1.1	Ribosomal protein S5 family	2964 / 10	1, 1, 1, 1	1, 0
	5699	1.2.1.1.1.1	UGT72E3*	1446 / 1	1, 1, 1, 1	1, 0
	5831	1.2.1.1.1.1	PM* choline transporter family	1449 / 3	1, 1, 1, 1	1, 0
	0-1050	1.2.1.1.1.1	zinc finger protein-related	5328 / 4	1, 1, 1, 1	1, 0
Cru	2442	1.1.6.1.1.1	AGAMOUS-like 87 (AGL87)	492 / 1	5 (4), 5, 1, 1	5, 0
	3071	1.1.5.1.1.1	RNA-binding (RRM/RBD/RNP motifs) family	546 / 4	4 (2), 3, 4, 3	3, 0
	4286	1.1.3.1.1.1	NMNAT*	639 / 8	2, 2, 2, 2	0, 2
	4551	1.1.3.1.1.1	LOB domain-containing protein 41 (LBD41)	795 / 2	2, 2, 2, 2	0, 2
	4140	1.1.3.1.1.1	myb domain protein 95 (MYB95)	852 / 3	2, 2, 1, 1	0, 2
	4421	1.1.3.1.1.1	hydroxycinnamoyl transferase (HCT)	1296 / 2	2 (2), 2, 1, 1	0, 2
	5691	1.1.2.1.1.1	SRP-related	1821 / 13	1, 1, 1, 1	0, 1
	5251	1.1.2.1.1.1	Calcium-binding EF-hand family	639 / 1	1, 1, 1, 1	1, 0
Esa	**2516**	**1.1.1.6.1.1**	**SALT-TOLERANCE 32 (SAT32)**	**1317 / 13**	**5 (3), 4, 3, 3**	**0, 4**
	4071	1.1.1.3.1.1	Cyclin A2;4 (CYCA2;4)	1401 / 10	2, 2, 2, 2	0, 2
	3945	1.1.1.3.1.1	prenylated RAB acceptor 1.E (PRA1.E)	621 / 1	2, 2, 1, 1	2, 0
	4237	1.1.1.3.1.1	RNA-DIRECTED DNA METHYLATION 1 (RDM1)	480 / 2	2, 2, 1, 1	2, 0
	4314	1.1.1.3.1.1	SPFH/Band 7/PHB domain-containing	1077 / 9	2 (2), 2, 1, 1	0, 2
	4608	1.1.1.2.1.1	MAPKKK5*	2154 / 11	1, 1, 1, 1	0, 1
	4633	1.1.1.2.1.1	KOKOPELLI (KPL)	1473 / 3	1, 1, 1, 1	0, 1
	4867	1.1.1.2.1.1	TERMINAL FLOWER 1 (TFL1)	534 / 4	1, 1, 1, 1	1, 0
	5499	1.1.1.2.1.1	xylem cysteine peptidase 1 (XCP1)	1068 / 4	1, 1, 1, 1	1, 0
	5701	1.1.1.2.1.1	NAC domain containing protein 6 (NAC6)	870 / 3	1, 1, 1, 1	1, 0
Sir	3061	1.1.1.1.5.1	cytochrome P450 (CYP78A10)	1620 / 2	4, 3, - , -	3, 0
	3400	1.1.1.1.4.1	amino acid permease 3 (AAP3)	1431 / 7	3, 2, - , -	0, 2
	3964	1.1.1.1.3.1	PALE CRESS (PAC)	603 / 5	2, 1, - , -	1, 1
	5051	1.1.1.1.2.1	GLOBULAR ARREST1 (GLA1)	1707 / 8	1, 1, - , -	0, 1
	5943	1.1.1.1.2.1	DROUGHT SENSITIVE 1 (DRS1)	1662 / 4	1, 1, - , -	0, 1
	6060	1.1.1.1.2.1	YUCCA5 (YUC5);	1281 / 3	1, 1, - , -	1, 0
Spa	3944	1.1.1.1.1.3	Endomembrane protein 70 ;	1779 / 7	2, 2, 2, 2	1, 1
	4729	1.1.1.1.1.2	UQCRX* -like family	204 / 3	1, 1, 1, 1	0, 1
	4815	1.1.1.1.1.2	zinc transporter 3 precursor (ZIP3)	1029 / 3	1, 1, 1, 1	1, 0
	5521	1.1.1.1.1.2	20S proteasome alpha subunit G1 (PAG1)	750 / 10	1, 1, 1, 1	0, 1
	6085	1.1.1.1.1.2	WRKY DNA-binding protein 72 (WRKY72)	1659 / 4	1, 1, 1, 1	0, 1
	1764-1	1.1.1.1.1.2	diacylglycerol kinase 5 (DGK5)	1482 / 13	1, 1, 1, 1	0, 1
	403-2	1.1.1.1.1.2	Alba DNA/RNA-binding protein	396 / 5	1, 1, 1, 1	0, 1

[a] A partial list of OrthNets including the most Tr copies with complete ORFs (i.e. ORF size within ±20% in proportion compare to the CL copy) and with RNA-seq expression evidence. The complete list is in Dataset S3.
[b] Number of nodes for each genome, sorted alphabetically, i.e. Number of nodes for Aly.Ath.Cru.Esa.Sir.Spa
[c] If multiple CL copies exist, mostly due to tandem duplication (TD), we chose the one with the longest ORF.
[d] Number of Total Tr copies with Tr copies tandem duplicated in parentheses, Tr copies with complete ORF (cORF), Tr copies with RNA-seq expression evidence (exp), and Tr copies with both cORF and exp (cORF & exp)
[e] g+i, the homologous genome segment (HGS) includes both gene and intergenic regions; go, gene-only *tr-d*, same as in Fig. 6D. The sum of g+i and go sets is equal to the "Complete duplication" set in Fig. 6.

* Abbreviations in annotation: PAI3, phosphoribosylanthranilate isomerase 3; HPA1, histidinol phosphate aminotransferase 1; UGT72E3, an UDPG:coniferyl alcohol glucosyltransferase; PM, plasma membrane; NMNAT, nicotinate/nicotinamide mononucleotide adenyltransferase; MAPKKK5, mitogen-activated protein kinase kinase kinase 5; UQCRX, ubiquinol-cytochrome C reductase

Table S6. List of OrthNets including *tr-d* events shared by *E. salsugineum* (Esa) and *S. parvula* (Spa)[a]

Category	OrthNet ID (ON_)	Node count pattern [b]	Annotation	Genomes	CL copy [c] ORF length (nt) / # exons	Total, cORF, exp, cORF & exp [d]
Ind-parallel	3530	1.1.1.3.1.2	Nucleotidylyl transferase superfamily	Esa	1200 / 12	2, 2, 2, 2
				Spa	1209 / 12	1, 1, 0, 0
	0-0850	1.1.1.2.1.3	Zinc-finger domain, monoamine-oxidase A repressor R1	Esa	1410 / 10	1, 1, 0, 0
				Spa	1656 / 13	1, 1, 0, 0
	3452	1.1.1.3.1.2	novel cap-binding protein (NCBP)	Esa	678 / 6	2, 0, 2, 0
				Spa	678 / 6	1, 1, 1, 1
	3602	1.1.1.2.1.3	calcium-dependent protein kinase 1 (CDPK1)	Esa	1638 / 7	1, 0, 1, 0
				Spa	1464 / 8	2, 1, 0, 0
	4248	1.1.1.2.1.2	general regulatory factor 2 (GRF2)	Esa	783 / 4	1, 0, 1, 0
				Spa	783 / 4	1, 1, 1, 1
	4216	1.1.1.2.1.2	ABA INSENSITIVE 1 (ABI1)	Esa	1320 / 4	1, 0, 1, 0
				Spa	975 / 4	1, 0, 0, 0
	4253	1.1.1.2.1.2	inositol polyphosphate 5-phosphatase 11 (5PTASE11)	Esa	933 / 6	1, 0, 1, 0
				Spa	1008 / 7	1, 0, 0, 0
	3427 [e]	1.1.1.2.2.2	embryo defective 2191 (emb2191)	Esa	654 / 5	1, 1, 1, 1
				Spa	561 / 7	1, 1, 1, 1
Tr-*cl*	170-3	1.1.1.2.1.2	basic helix-loop-helix (bHLH) DNA-binding superfamily	Esa	1092 / 7	1, 1, 1, 1
				Spa	1077 / 7	1, 1, 1, 1
	8-07	1.1.1.2.1.2	polygalacturonase 4 (PGA4)	Esa	1266 / 4	1, 1, 1, 1
				Spa	1266 / 4	1, 1, 0, 0
	3919	1.1.1.2.1.2	unknown protein	Esa	360 / 1	1, 1, 0, 0
				Spa	357 / 1	1, 1, 1, 1
	3913	1.1.1.2.1.2	SNARE-like superfamily	Esa	456 / 6	1, 1, 1, 1
				Spa	408 / 5	1, 1, 0, 0
	4390	1.1.1.2.1.2	RING/U-box superfamily	Esa	426 / 1	1, 0, 1, 0
				Spa	375 / 2	1, 1, 0, 0
	4526	1.1.1.2.1.2	RING/FYVE/PHD zinc finger superfamily	Esa	1209 / 4	1, 0, 0, 0
				Spa	1188 / 5	1, 1, 1, 1
	2430 [e]	1.1.1.2.3.3	calcineurin B-like protein 10 (CBL10)	Esa	780 / 9	1, 1, 0, 0
				Spa	747 / 9	2, 2, 2, 2
	3847 [e]	1.1.1.2.2.2	NAC domain containing protein 58 (NAC058)	Esa	921 / 3	1, 1, 1, 1
				Spa	927 / 3	1, 1, 1, 1
	3474 [e]	1.1.1.2.2.2	P-loop nucleoside triphosphate hydrolases superfamily	Esa	3465 / 17	1, 1, 1, 1
				Spa	3492 / 17	1, 1, 0, 0
	240-2 [e]	1.1.1.2.2.2	Ribosomal protein L22p/L17e family	Esa	528 / 7	1, 1, 0, 0
				Spa	528 / 7	1, 1, 0, 0
	3310 [e]	1.1.1.2.3.2	CRUMPLED LEAF (CRL)	Esa	783 / 10	1, 1, 1, 1
				Spa	810 / 9	1, 1, 1, 1
	0-0834 [e]	1.1.1.2.2.2	photosystem II reaction center W (PSBW)	Esa	402 / 2	1, 1, 1, 1
				Spa	402 / 2	1, 1, 0, 0
	1066-1 [e]	1.1.1.2.3.3	phosphorylcholine cytidylyltransferase (CCT1)	Esa	996 / 8	1, 1, 1, 1
				Spa	996 / 8	2, 1, 1, 1

[a] The complete list for all pairs of genomes is in Dataset S3.

[b] Number of nodes for each genome, sorted alphabetically, i.e. Number of nodes for Aly.Ath.Cru.Esa.Sir.Spa

[c] If multiple CL copies exist, mostly due to tandem duplication (TD), we chose the one with the longest ORF

[d] Number of Total Tr copies with Tr copies tandem duplicated in parentheses, Tr copies with complete ORF (cORF), Tr copies with RNAseq expression evidence (exp), and Tr copies with bot cORF and exp (cORF & exp)

[e] These OrthNets showed Lineage II-specific *tr-d* events, with all additional Tr copies in Sir having incomplete ORFs, essentially resulting in copy number increments specific to Esa and Spa only.

A

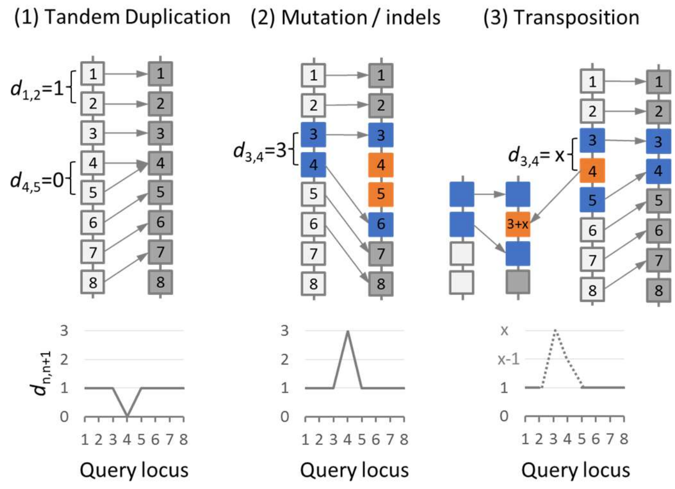

B

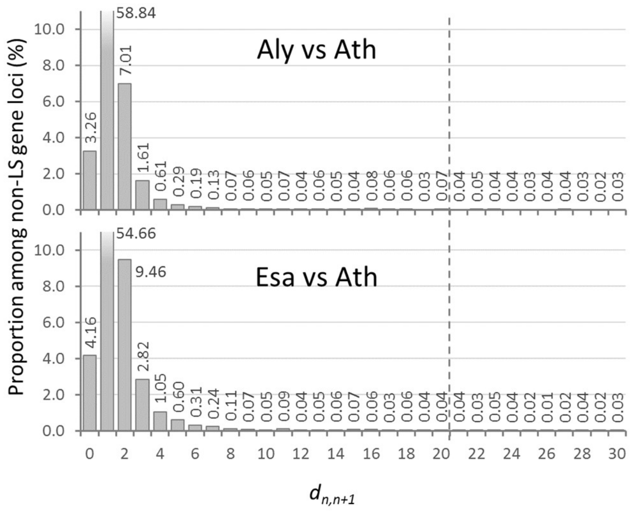

C

$d_{n,n+1}$	Proportion among non-LS gene loci (%)				
	Aly-Ath	Cru-Ath	Esa-Ath	Sir-Ath	Spa-Ath
0[a]	3.26	4.31	4.16	4.54	2.88
1[b]	58.84	61.51	54.66	55.36	61.22
2~20	10.58	15.91	15.24	17.36	16.67
>20	7.98	5.27	6.91	6.60	5.10
Diff Chr[c]	19.34	12.99	19.02	16.19	14.14

D

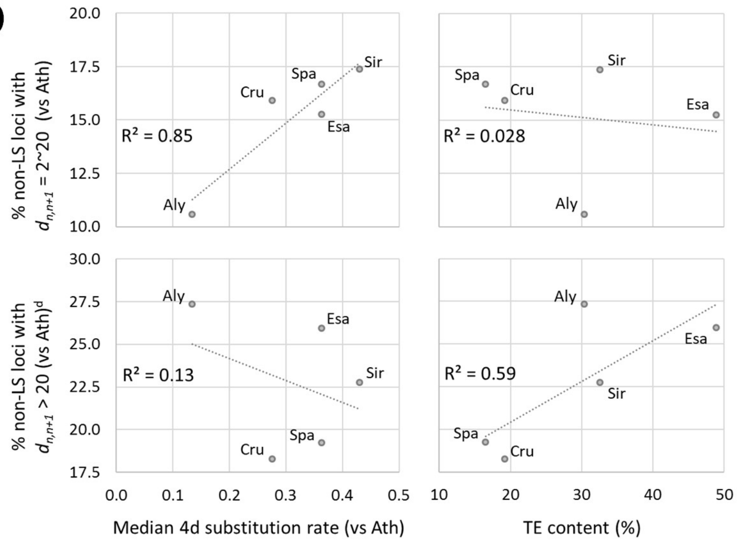

E

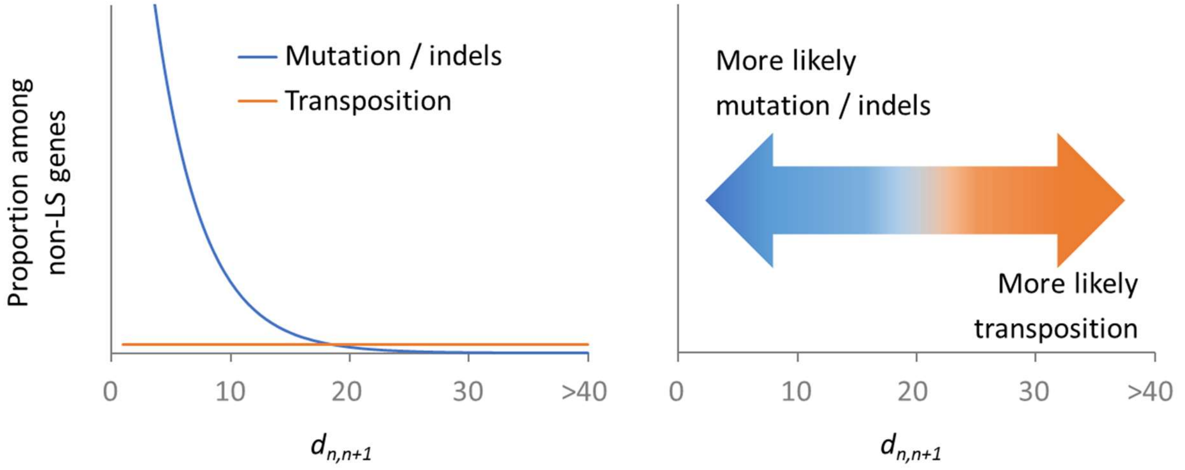

Figure S1. Erosion of co-linearity between Brassicaceae (crucifer family) genomes (legend in the next page)

Figure S1. Erosion of co-linearity between Brassicaceae (crucifer family) genomes

(A) Each locus in the query genome (light gray) is paired with an arrow to its most homologous counterpart locus ("best-hit") in the target genome (gray), after numbering all loci in both genomes based on their genomic positions. The difference in best-hit loci positions between the query locus at position *n* and its neighbor at position *n+1* ($d_{n,n+1}$) is 1 when the query locus and its best-hit in the target are perfectly co-linear, while a tandem duplication unique to the query genome results in $d_{n,n+1} = 0$ (1). Co-linearity is disrupted by mutations and indels in the neighboring loci (2), as well as a gene transposition event (3). Gene loci that show disrupted co-linearity due to mutations and indels in neighboring loci are colored in blue, while orange loci represent those transposed and completely lost the evidence of a common ancestry (i.e., synteny) in their respective genomic neighborhoods.
(B, C) The distribution of $d_{n,n+1}$ with either *A. lyrata* (Aly), *C. rubella* (Cru), *E. salsugineum* (Esa), *S. irio* (Sir), and *S. parvula* (Spa) as the query and the *A. thaliana* (Ath) genome as the target. Aly-Ath and Esa-Ath pairs are shown as exemplary histograms in B. Results for all pairs are summarized in C.
(D) The proportion of gene loci with $d_{n,n+1}$ ranging from 2 to 20 showed a higher correlation with the median four-degenerated (4d) substitution rate (upper panels). Contrastingly, the proportion of those with $d_{n,n+1}$ larger than 20 or their best-hits found in a different target chromosome was better correlated with the overall transposable element (TE) contents (lower panels).
(E) A schematic representation of two underlying models, mutations and indels in neighboring loci (blue) and transposition (orange), to explain the pattern of co-linearity erosion observed in (B). Co-linearity around a gene locus can be disrupted by mutations and indels occurring in the adjacent loci. Such mutations and indels involve mostly small numbers of neighboring loci and do not completely remove synteny, as exemplified by blue loci in (A). In this model, higher $d_{n,n+1}$ requires multiple mutations and indels co-occurring in the same neighborhood and expected to be rare (left panel, blue line). Contrastingly, transposition events, as depicted in the orange-colored loci in (A), can explain a gene movement to a distant locus or to a different chromosome, resulting in the loss of synteny. Short sequence motif pairs required for a transposition by the double strand break repair by non-homologous end-joining (DSB-NHEJ) model are virtually ubiquitous throughout the genome. Hence, transposition can happen between any loci at a uniform frequency regardless of $d_{n,n+1}$ (left panel, orange line). Combined together, the co-linearity erosion with smaller $d_{n,n+1}$ tends to be due to mutations and indels in the neighboring loci (right panel, blue arrow), while a larger $d_{n,n+1}$ is most likely a result of transposition events (right panel, orange arrow). In the observed data (B), the exponential decay of non-LS gene proportion came to a plateau at approximately $d_{n,n+1} > 20$ and we postulate that most genes in this region are transposed. We used this value as the parameter for the maximum gap between co-linear loci-in-chain (i.e. *G*=20) for the CLfinder analysis between pairs of Brassicaceae genomes.

[a] The query locus involved in a tandem duplication event unique to the query genome.
[b] The query locus co-linear with its best-hit without any modification in gene order.
[c] The best-hit of the query locus *n* is located in a different chromosome ("Diff Chr") in *A. thaliana*, compared to the best-hit of its immediate neighbor locus *n+1*.
[d] Includes loci with $d_{n,n+1} > 20$ as well as the "Diff Chr" indicated in (*C*)

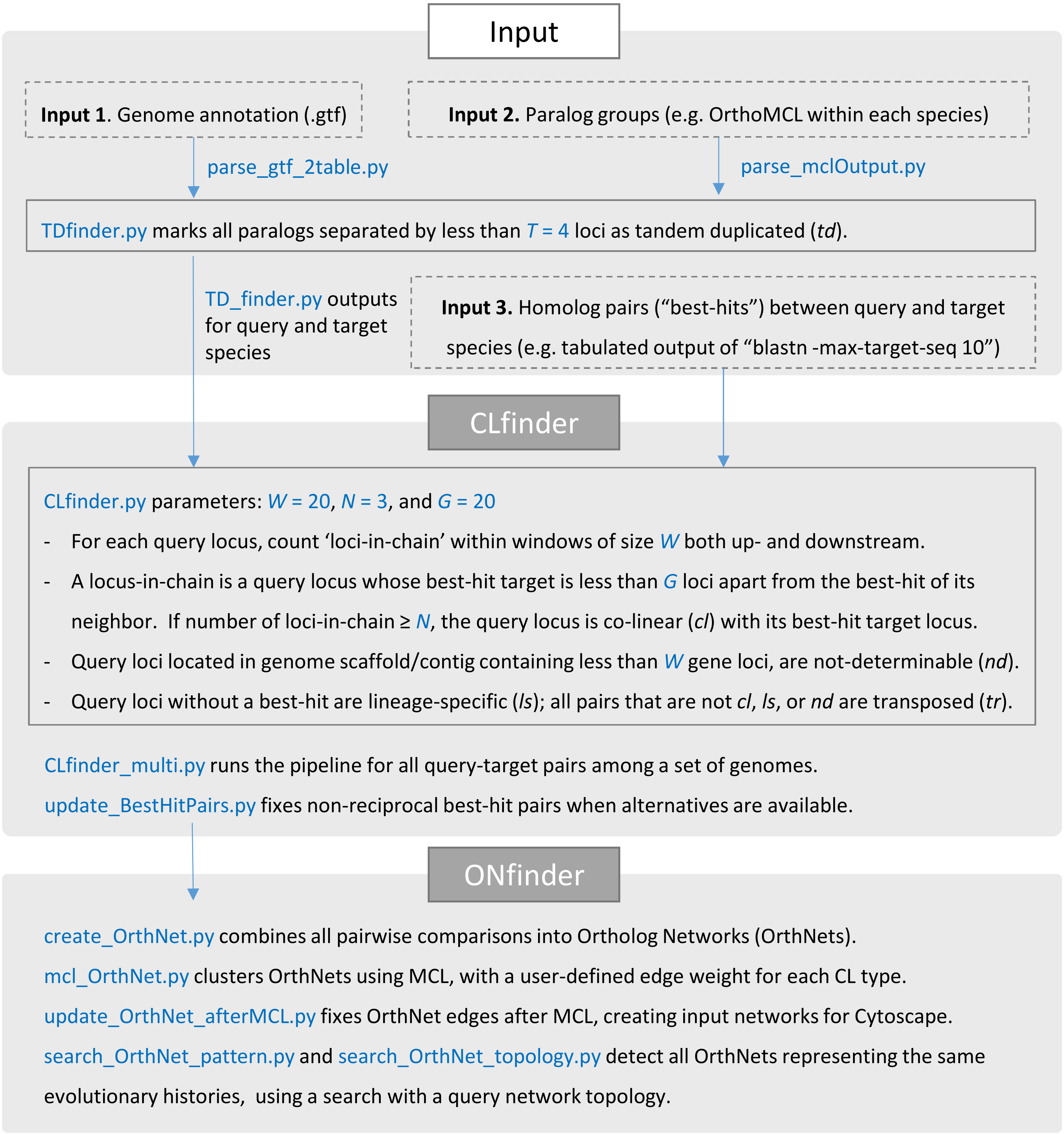

Figure S2. A detailed flowchart of the CL_finder-OrthNet pipeline.

Parameters to adjust the sensitivity and stringency of co-linearity detection are as follow: the window size (*W*), the number of co-linear loci-in-chain threshold (*N*), the maximum gap allowed between co-linear loci-in chain (*G*), and maximum loci difference between tandem duplicates (*T*). Listed parameters are values used for the analysis of the six Brassicaceae genomes in the present study. All scripts and a step-by-step guide are available at the project GitHub page (https://github.com/ohdongha/CL_finder).

	(1)	(2)	(3)	(4)				(5)
Spa geneID	Spa locusID	Spa_PG	Spa_TDid	Ath geneID	Ath locusID	Ath_PG	Ath_TDid	Spa-Ath_CL 20.3.20
…	…	…	…	…	…	…	…	…
Sp2g01320	4704	PG01085	-	AT1G63220	5238	PG01514	-	cl_u3d3
Sp2g01330	4705	PG03062	-	AT1G63210	5237	PG02567	-	cl_u3d3
Sp2g01340	4706	PG01299	Spa|PG01299_TD01	AT1G63190	5233	PG00454	Ath|PG00454_TD01	cl_u3d3
Sp2g01350	4707	PG02334	Spa|PG02334_TD01	na	na	na	na	ls
Sp2g01360	4708	PG01299	Spa|PG01299_TD01	AT1G63190	5233	PG00454	Ath|PG00454_TD01	cl_u3d3
Sp2g01370	4709	na	-	AT3G19910	13789	PG01811	-	tr
Sp2g01380	4710	PG02334	Spa|PG02334_TD01	na	na	na	na	ls
Sp2g01390	4711	PG00325	-	AT1G63180	5232	PG00362	-	cl_u3d3
Sp2g01400	4712	PG00604	-	AT1G67390	5677	PG00693	-	tr
Sp2g01410	4713	PG00353	-	AT1G63170	5231	PG00427	-	cl_u3d3
Sp2g01420	4714	na	-	na	na	na	na	ls
Sp2g01430	4715	PG01143	-	AT1G63160	5230	PG00642	-	cl_u3d3
Sp2g01440	4716	PG00106	Spa|PG00106_TD02	AT1G63140	5228	PG00161	-	cl_u3d3
Sp2g01450	4717	PG00106	Spa|PG00106_TD02	AT1G63140	5228	PG00161	-	cl_u3d3
Sp2g01460	4718	PG00415	-	AT1G63120	5226	PG00441	-	cl_u3d3
Sp2g01470	4719	PG00066	-	AT1G12740	1260	PG00065	-	tr
Sp2g01480	4720	na	-	AT1G63110	5225	PG02480	-	cl_u3d3
Sp2g01490	4721	PG00091	-	AT1G63100	5223	PG00108	-	cl_u3d3
Sp2g01500	4722	PG00617	-	AT1G63090	5222	PG00831	-	cl_u3d3
Sp2g01510	4723	PG03063	-	AT1G63060	5219	PG03035	-	cl_u3d3
Sp2g01520	4724	PG02539	-	AT1G63050	5216	PG02358	-	cl_u3d3
Sp2g01530	4725	PG00296	Spa|PG00296_TD02	AT1G12630	1249	PG00309	Ath|PG00309_TD01	tr
Sp2g01540	4726	PG00296	Spa|PG00296_TD02	AT1G12630	1249	PG00309	Ath|PG00309_TD01	tr
Sp2g01550	4727	PG00296	Spa|PG00296_TD02	AT1G63030	5215	PG00309	-	cl_u3d3
Sp2g01560	4728	PG03064	-	AT1G63020	5214	PG03100	-	cl_u3d3
…	…	…	…	…	…	…	…	…

Figure S3. An example of the CLfinder process, comparing *S. parvula* (Spa) and *A. thaliana* (Ath) as the query and target genome, respectively.

The "TDfinder.py" script (Fig. S1) prepares the genome annotations (Fig. S1, Input 1) for the CLfinder process as follows: (1) Assign numerical "Locus ID (locusID)" based on gene order; (2) Add Paralog Group (PG) information (Fig. S1, Input2) to each locus; (3) Identify tandem duplication based on Locus ID and PG information, i.e. all loci that belong to the same PG and separated by the same or less than max_TD_loci_dist (T = 4) are considered tandem duplicated and given a unique tandem duplication ID (TDid). Afterward, the "CLfinder.py" script (Fig. S1) compares query and target genomes as follows: (4) For each locus in the query genome, identify the most homologous counterpart, or best-hit, among the target genome loci based on Input 3 (Fig. S1). Locus ID, PG, and TDid for the best-hit locus is also added; (5) Identify co-linearity relationship between each query locus and its best-hit in the target genome as described in Methods. See also the legend and footnote of Supplementary Dataset S1 for more detail.

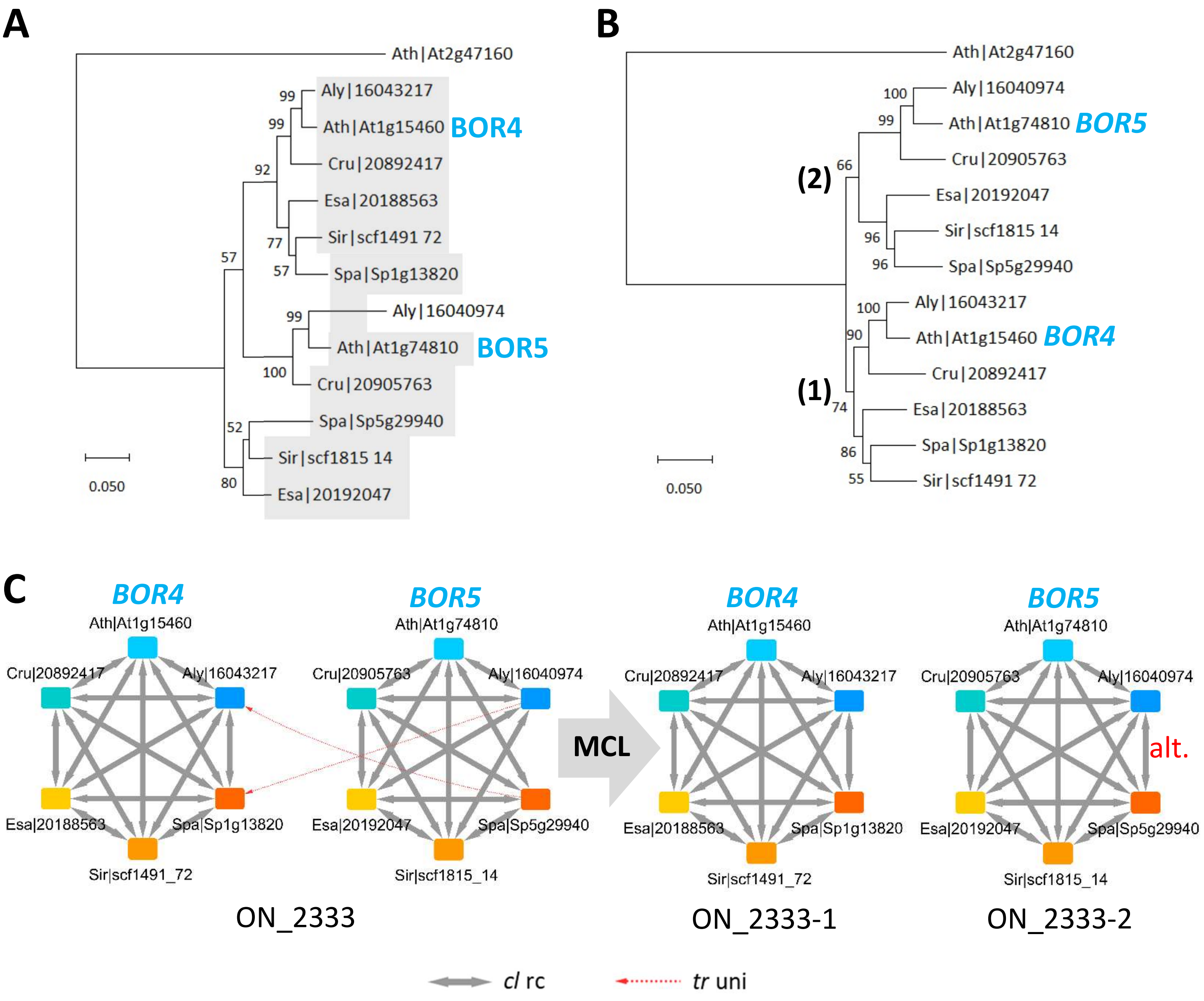

Figure S4. An example of Markov clustering (MCL) process to resolve OrthNets containing outparalogs

(**A**) In the tree based on protein sequences of BORON TRANSPORTER4 (BOR4; At1g15460) and BOR5 (At1g74810) orthologs, the two ortholog groups were not separated and OrthoFinder grouped BOR4 and BOR5 orthologs in a single cluster (shaded in gray). Contrastingly, the tree based on ORF nucleotide sequences (**B**) identified two distinct branches, each including *BOR4* (1) and *BOR5* (2) orthologs for all six species, respectively. This indicates that *BOR4* and *BOR5* are out-paralogs duplicated before the divergence of the six species. The genomic positional information also supported this notion, as CLfinder-Orthnet identified *BOR4* and *BOR5* networks as two distinct hexagons consisting reciprocally co-linear (*cl* rc) edges (**C**). CLfinder-OrthNet initially identified *BOR4* and *BOR5* orthologs in one OrthNet (ON_2333). This was the result of *BOR5* orthologs in *Arabidopsis lyrata* (Aly|16040974) and *Schrenkiella parvula* (Spa|Sp5g29940) pairing with *BOR4* orthologs in their counterpart species as Best-Hit pairs, connecting the two co-linear hexagons with two unidirectional transposition (*tr* uni) edges. Such an incongruence between sequence homology and co-linearity, where Best-Hit pairs occur between orthologs of out-paralogs rather than their co-linear orthologs, often results in grouping networks derived from out-paralog loci into a single OrthNet. We used MCL to separate such OrthNets containing out-paralogs, as exemplified by the separation of the *BOR4* and *BOR5* OrthNets after MCL, as ON_2333-1 and ON_2333-2, respectively. The edge indicated with "alt." in ON_2333-2 connects alternative best-hit pairs identified after the MCL separated the two networks. See Methods for the detailed process to find the alternative edge after MCL. Trees in (**A**) and (**B**) were inferred based on maximum likelihood, with the percent bootstrap support from 500 iterations shown for each branch. BOR1 (At1g47160) was used as an outgroup.

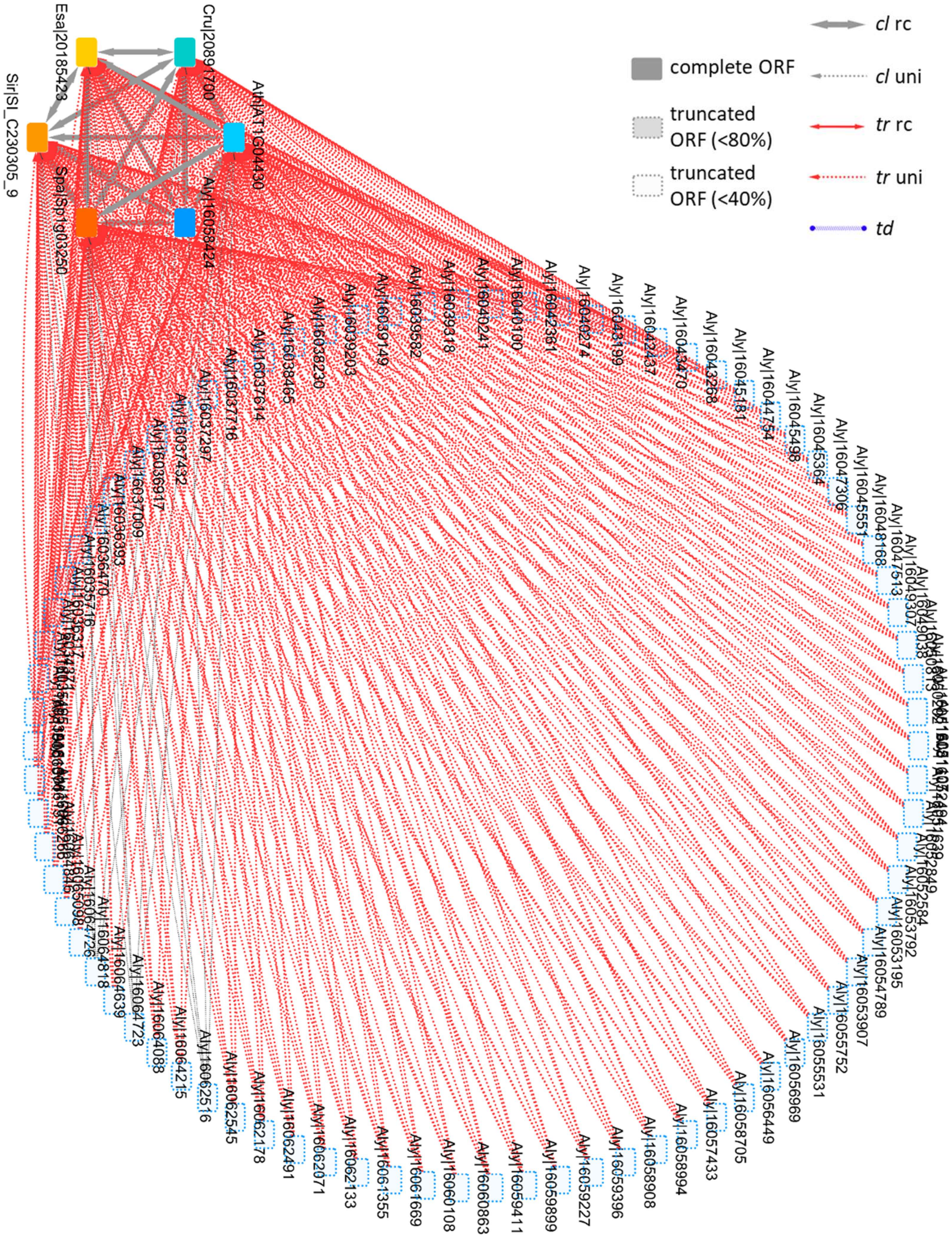

Figure S5. An example of *A. lyrata* (Aly)-specific *tr-d* events with a large number of duplicated copies (OrthNet ID: On_40). Node and edge properties are as described in Fig. 4A. In this OrthNet, more than 90% of all duplicated Tr copies included truncated ORFs of 150~516nt that were partial sequences of a Helitron transposon. None of the Tr copies showed evidence of expression (Dataset S3). We excluded this particular OrthNet from the analysis of lineage-specific *tr-d* events (Figures 4 and 6) based on high similarity to a known TE. OrthNet ON_40 was by far the largest showing lineage-specific *tr-d.* The second largest OrthNet with a lineage-specific *tr-d* event, ON_1227, contained only ten Tr copies (Dataset S3).

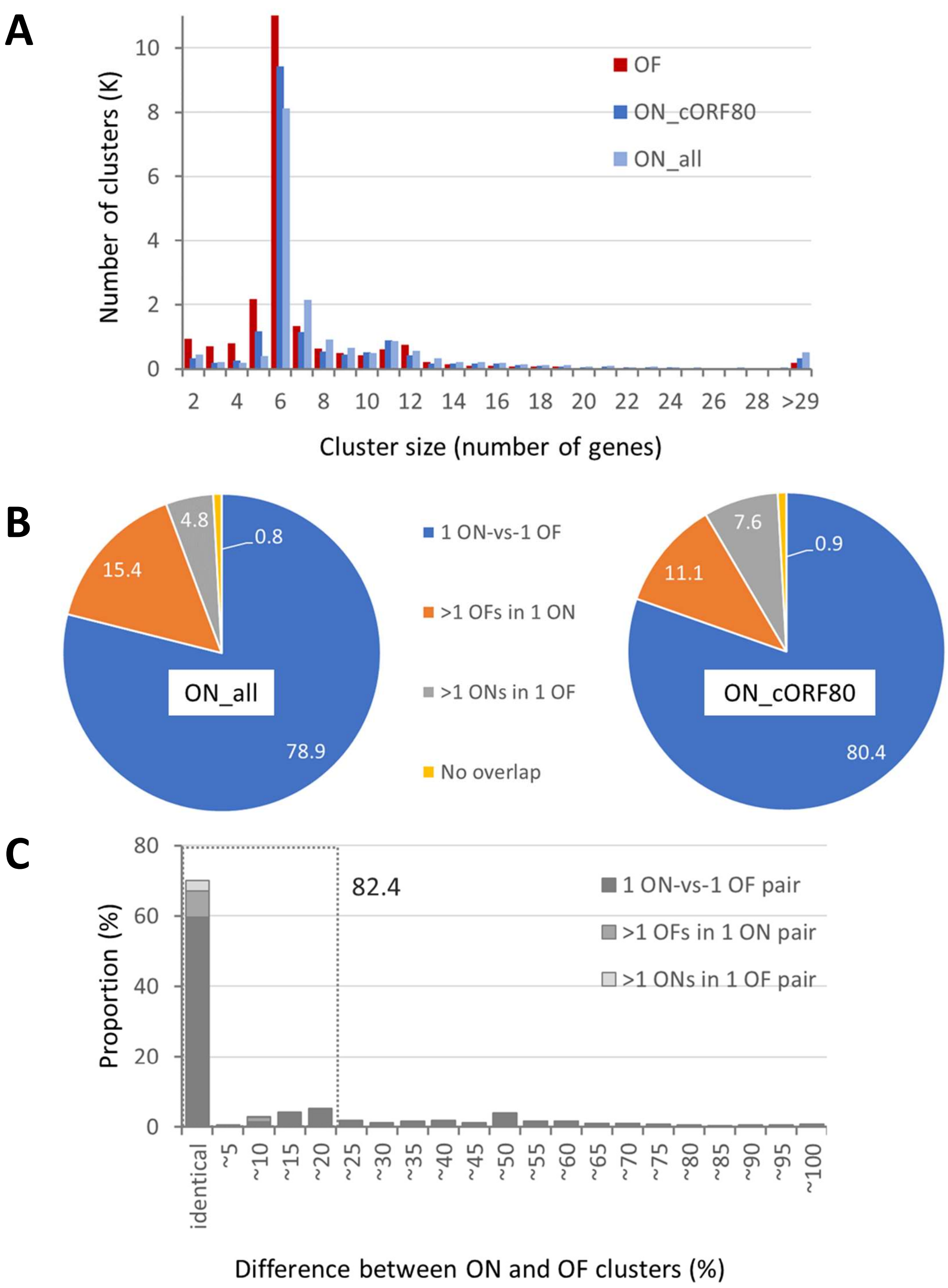

Figure S6. Comparison of orthologous gene clusters identified by OrthNet (ON) and OrthoFinder (OF).

(A) Comparison of size distributions between orthologous gene clusters detected by OrthoFinder (OF) and OrthNet (ON). For OrthNet clusters, either all nodes (ON_all) or nodes containing complete ORFs, i.e. ORFs larger than 80% of the median of their orthologous best-hit loci (ON_cORF80) were shown. The Y-axis is the number of clusters in thousands. **(B)** Total 17,342 ON clusters were paired with OF clusters sharing the largest number of genes, either one-by-one (1 ON-vs-1 OF), multiple OF clusters included in one ON cluster (>1 OFs in 1 ON), or multiple ON clusters in one OF cluster (>1 ONs in 1 OF). **(C)** A histogram showing differences between ON and OF clusters in total 16,345 ON-OF cluster pairs. ON clusters are from ON_cORF80 in panels A and B. The differences were calculated as (ON ∪ OF - ON ∩ OF) / (ON ∪ OF) (%), where ON and OF are either a single cluster (1 ON and 1 OF) or the sum of multiple clusters (>1 OFs and >1 ONs) in each pair. Total 70.1% and 82.4% of ON-OF pairs have identical clusters and clusters with less than 20% differences (marked by the dashed box), respectively.

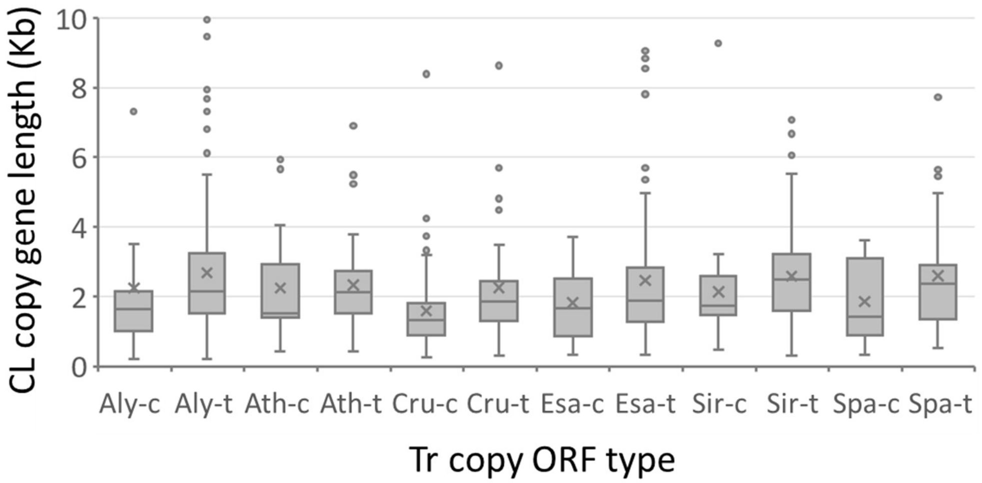

Figure S7. Comparison of CL copy gene lengths between Tr copies with complete (-c) and truncated (-t) ORFs in six Brassicaceae genomes

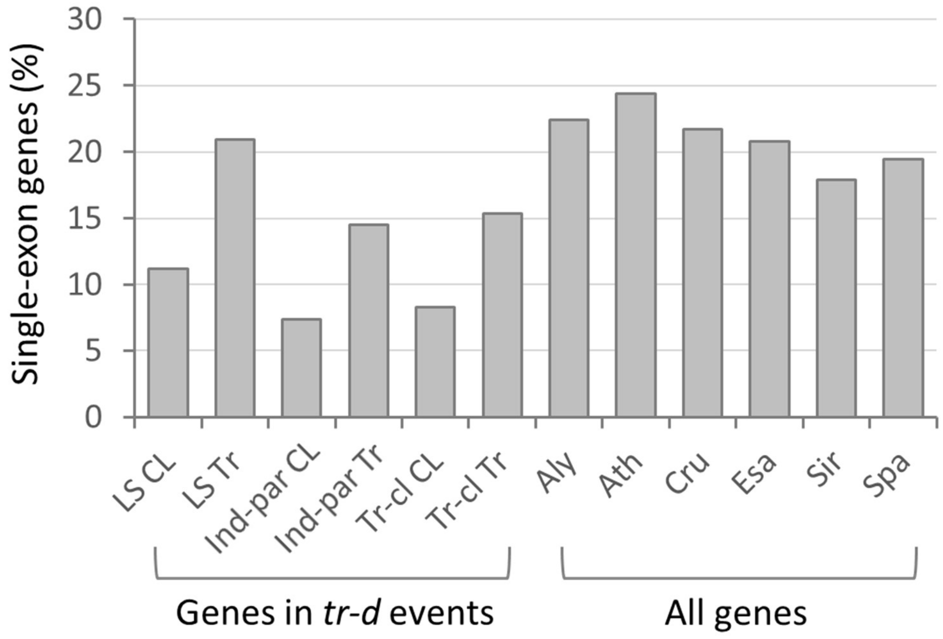

Figure S8. Proportion of single-exon genes among CL and Tr copies in lineage-specific (LS) and shared *tr-d* events, compared to all genes in six Brassicaceae genomes. See Fig. 5A and 6A for the detailed definition of CL and Tr copies, as well as Ind-parallel and Tr-*cl* categories

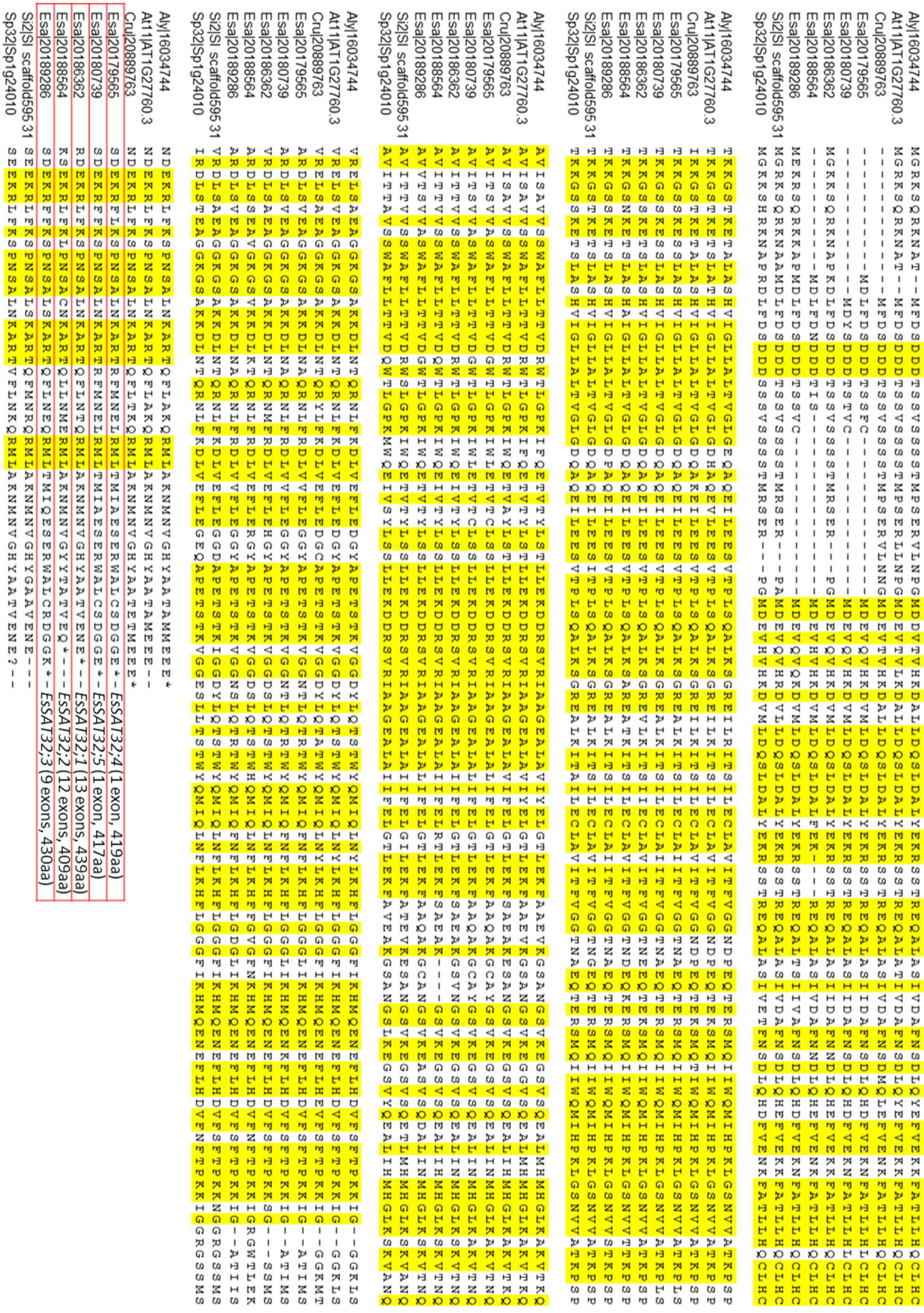

Figure S9. An alignment of deduced amino acid sequences for Brassicaceae *SALT TOLERANCE 32* (*SAT32*) genes
