# Supplementary material for: Landscape of gene transposition–duplication within the Brassicaceae family
Source: DNA Res. 2018 Oct 30;26(1):21–36. doi: 10.1093/dnares/dsy035 (PMC6379040; doi:10.1093/dnares/dsy035)
Supplement: Supplementary Data [file dsy035_supp.zip › dsy035-suppl_data/Oh_Dassanayake_Supplementary_Text_re2-revised.pdf]

Landscape of gene transposition-duplication within the Brassicaceae family

Dong-Ha Oh and Maheshi Dassanayake

Department of Biological Sciences, Louisiana State University, Baton Rouge, LA 70803

Correspondence: Dong-Ha Oh (ohdongha@gmail.com), Maheshi Dassanayake (maheshid@lsu.edu)

SUPPLEMENTARY TEXT

GLOSSARY 2

RESULTS & DISCUSSION 2

S1. Co-linearity erosion within Brassicaceae genomes 2

S2. Markov clustering to resolve large OrthNets into representations of individual loci 4

S3. Genes associated with lineage-specific transposition-duplication (*tr-d*) 6

S4. "Gene-only" duplications among *tr-d* events 7

S5. Distribution of *tr-d* events and transposable element (TE) contents 8

S6. Extremophyte crucifers constitutes a framework to study plant abiotic stress adaptation 8

S7. Genes associated with extremophyte-specific tr-d events 9

S8. *tr-d* events shared by extremophyte lineages and signatures of a convergent evolution 10

S9. Flexibility of the CLfinder-OrthNet pipeline 10

REFERENCES 11

GLOSSARY

Best-hit : the gene locus in the target genome most likely orthologous to a given gene in the query genome, in most cases the gene showing the highest sequence similarity among all genes in the target genome. CLfinder can also choose a best-hit based on mutual co-linearity among multiple best-hit candidates with comparable levels of sequence similarity.

Homologous Genome Segment (HGS): a stretch of genomic regions where sequence similarity can be detected among paralogous or orthologous loci. We detected HGSs between duplicated loci to determine "**gene-only (go)**" duplication, where sequence similarity was restricted to the gene region and not in the adjacent intergenic regions. A duplication that encompasses both gene and intergenic regions is indicated as a "**gene + intergenic (g+i)**" duplication.

OrthNet: a network that connects gene nodes to their best-hits in other genomes with co-linearity between nodes as edge properties. Edges can be **co-linear (*cl*)**, where the gene orders show a similarity that appears only explainable by a presence of a common ancestor or **transposed (*tr*)**, where the co-linearity is disrupted, losing any trace of common ancestry except for the sequence similarity within the gene. These edges can be either reciprocal or unidirectional. In addition, **tandem duplicated (*td*)** paralogs are connected with undirected edges. Each of the majority of OrthNets includes orthologs and tandem duplicated paralogs likely derived from a single ancestral locus.

Transposition-duplication (*tr-d*): an event where a gene locus (i.e., donor/source locus) duplicated and inserted a copy into an unrelated locus (i.e., acceptor/target locus) so that the common ancestry cannot be inferred except for coding sequence similarity. This is essentially the same as "Duplication mode IV" as defined by Freeling (2009). In an OrthNets representing a *tr-d* event, a donor locus is connected by reciprocal co-linear edges to its best-hit nodes, hence indicated as a "**CL copy**". An acceptor locus, or "**Tr copy**," is connected by unidirectional transposed edges to most of their best-hits. An OrthNet may include *tr-d* in multiple genomes either happened in each genome independently in parallel ("**Ind-parallel**") after their divergence, or in an ancestoral lineage, inferred by Tr copies from different genomes showing co-linearity ("**Tr-*cl***").

RESULTS & DISCUSSION

S1. Co-linearity erosion within Brassicaceae genomes.

We compared the gene orders in each of the Brassicaceae genomes except for *Arabidopsis thaliana* (query) with that of the *A. thaliana* genome (target) to analyze the degree of co-linearity erosion (Figure S1). We chose *A. thaliana* as the reference target because it has the best assembly among all plant genomes.

First, we assigned a numerical "Locus ID" to each non-transposable element (non-TE) protein-coding gene locus according to its position in the chromosome or genome scaffold. Then, using the Basic Local Alignment Search Tool for Nucleotides (BLASTN) (Camacho et al. 2009), we aligned all loci in the query

genome with their best BLASTN ($e<10^{-5}$) hits ("best-hit") among *A. thaliana* protein-coding genes. We excluded query loci without a BLASTN hit in the *A. thaliana* genome from the analysis.

Second, we designated the difference between Locus IDs of best-hits for query locus at position *n* and *n+1* as $d_{n,n+1}$ (Fig. S1A) and plotted the distribution of $d_{n,n+1}$ for each pair of genomes (Fig. S1B). The $d_{n,n+1}$ for the query locus *n* is expected to be one if the gene order is perfectly co-linear, zero if the query loci *n* and *n+1* are tandem duplicated (Fig. S1A, panel (1)), and larger than one for a gene located next to an insertion/deletion (indels) or an inversion of genomic segments (Fig. S1A, blue-colored loci in panel (2)). A gene transposition (*tr*) or transposition-duplication (*tr-d*) result in the best-hit to move to a position separated by any number (x) of loci, which may be on a different chromosome (Fig. S1A, orange-colored loci in panel (3)). The acceptor locus of a transposition or *tr-d* event (Fig. S1A, panel (3); Locus ID 3+x) may serve as the source of insertion (Fig. S1A, orange-colored loci in panel (2)). Deviation from the perfect co-linearity does not results in the loss of synteny (i.e., the evidence of a common ancestry) for genes adjacent to an indel or an inversion (Fig. S1A, blue-colored). On the other hand, a transposition and *tr-d* event moves the affected locus to a position where synteny can be no longer deduced (Fig. S1A, orange-colored).

If the co-linearity eroded over time, we would expect that the proportion of query loci with $d_{n,n+1} \neq 1$ will be larger in species diverged earlier from the target species. Indeed, $d_{n,n+1}$ distribution from the *A. lyrata*-vs-*A. thaliana* comparison (Fig. S1B, "Aly vs Ath") showed a greater percentage of loci with $d_{n,n+1} = 1$ than the *E. salsugineum*-vs-*A. thaliana* comparison (Fig. S1B, "Esa vs Ath"), reflecting the more recent divergence of *A. thaliana* from *A. lyrata* than from *E. salsugineum*. The proportion of query gene loci at a certain $d_{n,n+1}$ value rapidly decreased as $d_{n,n+1}$ increased from 1. This rapid decrease of the proportion of genes reached a plateau at relatively uniform non-zero values from $d_{n,n+1} = 20$ (Fig. S1B) to very large $d_{n,n+1}$ values up to thousands (data not shown).

For all pairs of genomes analyzed, we compared the proportion of genes showing displacements that were proximal (Fig. S1C, $d_{n,n+1} = 2\sim20$) and distal (Fig. S1C, $d_{n,n+1} > 20$ and displaced to a different chromosome) separately (Fig. S1C and D). The proportion of query loci with $d_{n,n+1} = 2\sim20$ showed a positive correlation ($R^2 = 0.85$; Fig. S1D, upper left panel) with the median four degenerate site (4d) substitution rate between all reciprocal best-hit pairs of the query and the target genomes. The median 4d substituation rate reflects the divergence time between the pair of genomes. In contrast, the proportion of query loci with $d_{n,n+1} > 20$ or whose best-hits found in a target chromosome different from their neighbors (Fig. S1C, $d_{n,n+1} > 20$ and "Diff Chr") did not show any positive correlation with the median 4d substitution rate (Fig. S1D, lower left panel). Instead, we found a moderate positive correlation ($R^2 = 0.59$) with the overall content of transposable element (TE) sequences (Fig. S1D, lower right panel). The proportion of loci with $d_{n,n+1} = 2\sim20$ was not correlated with the TE content ($R^2 = 0.028$; Fig. S1D, upper right panel).

This pattern suggested that two distinct models were responsible for eroding co-linearity (Fig. S1E). Proximal gene displacements (Fig. S1C, $d_{n,n+1} = 2\sim20$) were likely results of mutations and local indels and inversions occurred in the neighborhood of the locus (Fig. S1A, blue-colored loci). Genes affected by this

model become scarce as the $d_{n,n+1}$ increases (Fig. S1E, the blue graph and arrow) because larger $d_{n,n+1}$ values requires an indel encompassing larger numbers of neighboring loci or an accumulation of larger number of mutations in the neighborhood. On the other hand, distal gene displacements (Fig. S1C, $d_{n,n+1}$>20 or "Diff Chr") are more likely due to transposition whose frequency is on average uniform regardless of the $d_{n,n+1}$ (Fig. S1E, the orange graph and arrow). Mutations and indels accumulate over time, while the frequency of transposition events coincide with TE expansion, which may happen sporadically in each lineage regardless of the divergence time (Fig. S1D). We excluded all loci annotated as TE as well as lineage-specific gene models without detectable homology to a gene in the target genome (*A. thaliana*) from the analysis of co-linearity erosion. As a result, the distal displacement counts did not include transposition of TE genes. Still, TE genes can provide the anchor sequences for gene transposition through the non-homologous end-joining (NHEJ) repair of double strand break (DSB) and/or cause DSBs to initiate such an event (Wicker et al. 2010; Woodhouse et al. 2010). This explains the positive correlation between gene proportion with larger $d_{n,n+1}$ and the overall TE content of the genome (Fig. S1D).

The distal displacements were mostly due to single-gene transposition and transposition-duplication (*tr-d*) events (Supplementary Dataset S1), agreeing with a previous observation (Freeling 2009). Translocations of large chromosome-level segments also contributed, however, the effect was small. The six crucifer genomes consist of 24 large ancestral karyotype blocks reshuffled to five (*A. thaliana*), seven (*E. salsugineum*, *S. irio*, and *S. parvula*), and eight (*A. lyrata* and *C. rubella*) chromosomes (Lysak et al. 2006). The CLfinder module identifies borders of such blocks, and other smaller scale genomic segments that were either duplicated, deleted, inverted, or transposed, as *cl_end* types, which account for less than a few hundred loci in all pairwise comparisons combined (Methods and Supplementary Dataset S1).

We used the analysis of co-linearity erosion to determine a parameter for the CLfinder analysis on the six Brassicaceae genomes. We chose the $d_{n,n+1} = 20$, where the decay of gene proportion began to be more uniform (Fig. S1B), as the maximum gap between co-linear loci-in-chain (*G*) parameter. This was because genes showing co-linearity erosion with proximal displacement (Fig. S1A and E, blue colors) do not completely lose synteny and keep co-linearity towards at least one direction (Fig. S1A, blue loci). Neighboring loci whose best-hits were separated by less than *G* loci consisted co-linear loci-in-chain. A gene locus in the query genome is considered transposed compared to the target genome, only if it has less than *N* co-linear loci-in-chain (including itself) in a window of both up- and down-stream neighboring *W* loci (Figures S2 and S3).

S2. Markov clustering to resolve large OrthNets into representations of individual loci

The ONfinder module combined the CLfinder results into networks where a gene locus node was connected to their best-hits in all other genomes, with the co-linearity relationships between nodes as the edge property. The basic network was then enhanced by adding tandem duplicated (*td*) paralogs connected by undirected edges when present. We further attempted to divide the network into the smallest possible units,

using MCL with higher edge weights given to tandem duplicated and reciprocally co-linear edges. Therefore, the resulting OrthNets represented groups of orthologs likely derived from a single ancestral gene locus, with the addition of duplicated, transposed, and *tr-d* loci in varying combinations among the six Brassicaceae genomes. If duplicates were found equally in all six genomes, i.e. duplication dated before the divergence of the six genomes, they were represented in two or more separate OrthNets (see Figure S4 for an example), unless there was an incongruence between sequence homology and co-linearity as discussed below.

A challenge to the CLfinder-OrthNet approach, especially for plant genomes, is that often the co-linear loci do not necessarily include the most similar sequences, especially when each genome being compared include multiple paralogs. Such incongruence between co-linearity and sequence similarity results in a best-hit of a query locus found in a transposed location in the target genome, even when the co-linear location in the target genome also contained a gene showing homology with the query locus. This often resulted in an OrthNet containing orthologs from multiple out-paralog loci. To solve this problem, we designed the CLfinder module to accept multiple best-hit candidates for each query locus and scan for an alternative best-hit that give a reciprocal co-linear best-hit pair (See Methods). In addition, the OrthNet module employed MCL to separate large OrthNets into smallest possible units.

Figure S4 shows an example of OrthNets including orthologs from multiple out-paralogs due to an incongruence between sequence homology and co-linearity. Orthologs of *BORON TRANSPORTER4* (*BOR4*) and *BOR5* were unseparable in the tree based on protein sequences and included in a single cluster by OrthoFinder (Emms and Kelly 2015) (Fig. S4A). On the other hand, ORF nucleotide sequence (Fig. S4B) and genomic positions (Fig. S4C) indicate that they were outparalogs duplicated before the divergence of the six species. The initial OrthNet (Fig. S4C, ON_2333), where the reciprocally co-linear (Fig. S4C, "*cl* rc") hexagons of *BOR4* and *BOR5* orthologs were connected via unidirectional transposition edges (Fig. S4C, "*tr* uni"), was successfully separated by MCL (Fig. S4C, ON_2333-1 and ON_2333-2). Once the MCL separated the two OrthNets, the OrthNet module looked for alternative best-hit pairs among nodes in the newly separated OrthNets and connect them with an edge (e.g. the edge indicated by "alt." in Fig. S4C, ON_2333-2; See Methods for more details). Whether an edge was modified by MCL is recorded in the final CLfinder-OrthNet output (Supplementary Dataset S1).

For the MCL process on the six Brassicaceae genomes, we assigned a higher edge weight given in the order of *td* (1.5), reciprocal *cl* (1.2), unidirectional *cl* (0.6), reciprocal *tr* (0.5), and unidirectional *tr* (0.25) edges. Edge weights were decided with the following aims: (1) to separate networks of out-paralogs derived from multiple loci duplicated prior to the divergence of the six genomes (as exemplified in Fig. S4), while (2) keeping paralogs that underwent tandem duplication (*td*), transposition duplication (*tr-d*), and combinations of *td* and *tr-d*, together with the core set of co-linear orthologs in the same OrthNet.

OrthNets containing multiple outparalog loci were characterized by multiple complete or near-complete hexagons of reciprocally co-linear edges interconnected by small numbers of mostly unidirectional *tr* edges, as exemplified in Fig. S4C. Hence, for the aim (1), we assigned a smaller edge weight to an unidirectional *tr*

edge, compared to reciprocal *cl* edges. However, if the unidirectional *tr* edge weight is too small, there is a risk of losing in-paralogs duplicated by *tr-d*. As exemplified in Figures 4A, 5A, and 7A, paralog nodes duplicated by *tr-d* events tend to be connected to orthologs of five other species (i.e. *N*-1 species, where *N* is the number of all target species) with unidirectional *tr* edges. To prevent the MCL from separating *tr-d* paralogs into a separated OrthNet, we assigned the unidirectional *tr* edge a weight slightly larger than 1/5 (i.e. 1/(*N*-1)) of the weight given to the reciprocal co-linear edges. Once we decided the weights for the reciprocal *cl* edge (1.2) and the unidirectional *tr* edge (0.25), the weights for unidirectional *cl* and reciprocal *tr* edges were decided by considering one reciprocal edge as two unidirectional edges. Finally, the weight for non-directional *td* edge was empirically set at a value higher than that of a reciprocal *cl* edge but not too high to create another separated OrthNet including only tandem duplicated paralogs from one species. We tested this set of edge weights, as well as the MCL inflation parameter (I=1.2), on stress-related ion transporter paralogs we have identified as either in- or out-paralogs in a previous study (Oh et al. 2014), including *KEA1*/*2*, *CBL10;1*/*2*/*3*, and *HKT1;1/2/3* (in-paralogs), as well as *BOR4/5*, *NHX7*/*8*, *TIP2;2*/*3*, *KUP10*/*11*, and *VDAC2*/*5* (out-paralogs). The MCL process successfully separated all tested out-paralog loci into different OrthNets, while keeping in-paralogs in the same OrthNet.

Using this approach, we identified 86% of the total OrthNets including the same or less than 12 nodes in the current study (Fig. 3B). MCL has been used to identify orthologous groups based on a network graph with edges weighted for the sequence similarity (e.g. OrthoMCL and OrthoFinder) (Chen et al. 2006; Emms and Kelly 2015). Using different edge weights based on co-linearity relationships, the OrthNet module essentially attempts identification of orthologous gene groups based on genomic locations, instead of sequence similarities.

S3. Genes associated with lineage-specific transposition-duplication (*tr-d*)

In Table S5, the "Node count pattern" column, generated by the ONfinder module, indicate the copy numbers in each genome, in the alphabetic order of the species names, i.e. *A. lyrata* (Aly), *A. thaliana* (Ath), *C. rubella* (Cru), *E. salsugineum* (Esa), *S. irio* (Sir), and *S. parvula* (Spa). For each OrthNet, we listed the number of Tr copies with complete ORFs, with expression evidence, and with both (Table S5, "exp", "cORF", and "cORF & exp", respectively). We also included the number of tandem duplicated Tr copies in parenthesis followed by the total number of Tr copies and the number of gene-only and "g+i" types (as defined in Fig. 6D) among Tr copies in complete *tr-d* events. Among several notable high copy *tr-d* events specific to *A. lyrata*, *C. rubella*, *E. salsugineum*, and *S. irio* were OrthNets that included Tr copy paralogs encoding an unknown protein (ON_1227), a homeobox transcription factor AGAMOUS-like 87 (AGL87, ON_2442), an RNA-binding protein (ON_3071), a stress-signaling SALT-TOLERANCE32 (SAT32, ON_2516), a cytochrome P450 (ON_3061), and an amino acid permease 3 (ON_3400) (Table S5). We did not find lineage-specifc *tr-d* OrthNets with more than two Tr copies for either *A. thaliana* or *S. parvula*. This may be due to their compact genomes with lower repetitive sequence contents and also large numbers of *tr-d* events shared by *A. thaliana*-

A. lyrata and *S. parvula*-*S. irio* pairs, due to their phylogenetic proximity compared to other pairs in the target genomes (Fig. 5B and Dataset S3). The *E. salsugimeum* genome showed an expansion of loci related to stress responses such as *SAT32* (ON_2516) and *NAC6* (ON_5701) through *tr-d*, resulting in increased copy numbers with complete ORFs which were also expressed. In addition, *E. salsugimeum* contained *tr-d* events leading to higher copy number of reproduction and development-related loci, including *KOKOPELLI* (*KPL*) and *TERMINAL FLOWER 1* (*TFL1*). The *S. parvula* genome had increased copy numbers with complete ORFs and expression for loci encoding a zinc transporter (ZIP3; Table S5, ON_4815) and a WRKY transcription factor (WRKY72; Table S5, ON_6085).

We did not find a significantly over-represented gene ontology (GO) term among genes associated with lineage-specific *tr-d* in each genome tested, most likely due to the too small number of genes to be able to detect statistical enrichment. However, genes in lineage-specific *tr-d* events for all genomes combined showed enrichment of several GO terms (Supplementary Dataset S4). GO branches under "nitrogen compound metabolic process (adjusted P-value or P_{adj}=2.4 x10^{-4})" accounted for 172 out of total 812 lineage-specific *tr-d* genes with a GO annotation. Among them 121 were annotated with the child GO term "nucleic acid metabolic process (P_{adj}=0.020)" including 18 "DNA repair (P_{adj}=0.022)" genes. "Response to abiotic stimulus (P_{adj}=2.4x10^{-4})" included 93 genes, of which 37 belonged to "response to osmotic stress (P_{adj}=3.0x10^{-3})" while another 39 were "response to radiation (P_{adj}=0.020)". Other notable GO branches included "reproduction" (P_{adj}=0.012) and "post-embryonic development" (P_{adj}=0.014), which collectively included 88 genes. "Organelle organization (P_{adj}=2.9x10^{-7})" accounted for 77 gene loci, of which 31 and 13 were in child GO terms "chromosome organization (P_{adj}=1.1x10^{-4})" and "cytoskeleton organization (P_{adj}=0.031)", respectively. "Cell cycle (P_{adj}=1.1x10^{-4})" included 32 genes. The entire list of enriched GO terms is available as Supplementary Dataset S4.

S4. "Gene-only" duplications among *tr-d* events

Among Tr copy loci that retained complete ORFs in a *tr-d* event, a substantial fraction had the duplication start and end positions coincided with the boundaries of the coding region, resulting in sequence similarity found exclusively for the entire gene model but not in adjacent intergenic regions between the donor and duplicated loci. We described these as "gene-only" *tr-d* events (Fig. 6). Gene-only *tr-d* events may have had more time to lose sequence similarity outside of the coding sequence than the ones that retain similarity extended to intergenic regions. Indeed, we found gene-only *tr-d* events (Fig. 6D, "go") older than *tr-d* events where sequence similarities could be detected in both gene and intergenic regions (Fig. 6D, "g+i"), in all *tr-d* events either lineage-specific or independently occurred in parallel in two genomes (Fig. 6D, "LS" and "Ind-par"). For shared "Tr-*cl*" type *tr-d* events , the 4d substitution rates between duplicates (Fig. 6D, both "Tr-*cl*:g+i" and "Tr-*cl*:go") suggested that the majority of them were older than the divergence of the Brassicaceae genomes (Fig. 2D), consistent with the notion that these *tr-d* events were originated from the common ancestors of the genomes sharing them. Considering that intergenic regions are generally more prone to

mutations than genes, intergenic sequence similarity among duplicated paralog loci in these old shared *tr-d* events (Fig. 6D, "Tr-*cl*;g+i") may indicate purifying selection in intergenic regions suggestive of the presence of conserved non-coding regulatory elements.

S5. Distribution of *tr-d* events and transposable element (TE) contents

An intriguing pattern emerged when we considered the distribution of shared *tr-d* events in relation to overall TE contents (Table S4). TE may facilitate *tr-d* of non-TE genes by providing short sequence repeats that can act as anchors in DSB repair or TE activity may lead to initiation of DSB repair-mediated *tr-d* events (Wicker et al. 2010). Hence, we expect genomes with higher TE contents to contain more recent *tr-d* events. These recent *tr-d* will also have higher proportion of incomplete duplication and truncated ORF in Tr copy loci, since the duplications are more likely random before the functional duplicates are selectively retained over time.

Indeed, the three more TE-rich (>30% TE contents) genomes, *A. lyrata*, *E. salsugineum*, and *S. irio*, contained more lineage-specific *tr* and *tr-d* events, compared to the remaining three TE-depleted (<20% TE contents) genomes (Table S4). Interestingly, the highest number of lineage-specific *tr-d* events, majority of which occured recently after the divergence of the genome from its closest relative (Fig. 6C-D), was observed in *A. lyrata* rather than in *E. salsugineum*, although the latter contained the highest proportion of the genome annotated as TE (Table S4). *A. lyrata* and *S. irio* also included the smallest proportions of complete ORFs in Tr copy loci of lineage-specific *tr-d* events at 12.4% and 14.4%, respectively (Fig. 4D). Contrastingly, *E. salsugineum* showed a proportion of complete ORFs in Tr copy loci at 28%, comparable to other TE-depleted genomes (Fig. 4D).

One possible explanation is that *A. lyrata* and *S. irio* genomes may contain TEs that had expanded more recently than in the *E. salsugineum* genome. Both *A. lyrata* and *S. irio* have close relatives with low TE contents, *A. thaliana* and *S. parvula*, respectively. Between *A. lyrata* and *A. thaliana* genomes, TEs appeared to have expanded in *A. lyrata* rather than being lost in *A. thaliana*. The TE expansion in *A. lyrata*, as inferred by the expansion of recent long terminal repeat (LTR) retrotransposons starting at 1 million years ago (MYA) (Baidouri and Panaud 2013), is indeed more recent than the estimated *A. lyrata*-*A. thaliana* divergence at 3~5MYA (Hu et al. 2011). While beyond the scope of this study, it will be interesting to test whether the same is true for *S. irio* and *S. parvula* genomes, as well as comparing the age of LTRs in *E. salsugineum* with those in *A. lyrata* and *S. irio*. The percentage of loci showing distal best-hit displacement (Fig. S1C, $d_{n,n+1}$ >20 or "Diff Chr"), which reflect *tr-d* events, may show a better correlation with the content of more recently expanded TEs, than with total contents of sequences annotated as TE/repeats (Fig. S1D).

S6. Extremophyte crucifers constitutes a framework to study plant abiotic stress adaptation

The two extremophytes, *S. parvula* and *E. salsugineum*, share the capacity to survive under highly saline environments (Orsini et al. 2010). The habitats of these two extremophytes also provide additional challanges

dintinct to each species, multi-ion salt stresses for *S. parvula* (Oh et al. 2014) and combined salt and freezing stresses for *E. salsugineum* (Inan 2004; Amtmann 2009). Genomes of these two Lineage II extremophytes (Fig. 1) may contain signatures of adaptation both shared and unique to each species.

Comparisons of the genomes and transcriptomes of *E. salsugineum* and *S. parvula* with those of the model plant *A. thaliana* have identified a number of stress response-related orthologs showing differences in copy number, promoter sequences, and basal level expression strengths, as well as signatures of sub- and neo-functionalization among duplicates (Oh et al. 2010; Dassanayake et al. 2011; Ali et al. 2012; Wu et al. 2012; Oh et al. 2014). Functions of some of these modifications in stress adaptation have been confirmed by transgenic studies in the extremophyte species (Oh et al. 2009; Ali et al. 2012; Ali et al. 2016). However, pairwise comparison to a model species often has limited resolution in identifying candidate variations of interest. For example, comparison of *S. parvula* with the model *A. thaliana* genomes detected thousands of orthologous genes that show different duplication or gene transposition events (Table 1) and hundreds even after filtering for those showing variation in expression strengths (Oh et al. 2014). Given the high number of these candidates, often the hypothesis generation step has to focus on a subset of candidates with stronger prior functional evidence related to plant stress responses. The majority of these variations may reflect the more ancient divergence between the Brassicaceae Lineage I and II, rather than more recent local adaptations specific to an extremophyte. The CLfinder-OrthNet pipeline enables identifying lineage (monophyletic) or multiple lineages (polyphyletic or paraphyletic)-specific variations in a higher resolution, using a multi-genome comparative framework.

S7. Genes associated with extremophyte-specific *tr-d* events

Except for *SAT32*, OrthNets showing *E. salsugineum*- or *S. parvula*-specific *tr-d* did not include genes with known functions directly related to salt stress responses in *A. thaliana*. This is rather unsurprising considering that *A. thaliana* is naturally a salt-sensitive species and its response to salinity is mostly a symptom than a selective and strategic response aiming for tolerance or avoidance and, eventually, survival in extremely saline environments (Gong et al. 2005). OrthNets with *E. salsugineum*- or *S. parvula*-specific *tr-d* included loci encoding proteins annotated based on functional evidences in the model *A. thaliana*, involved in defense (Table S5, ON_4608, ON_6085, and ON_1764-1, encoding MAPKKK5, WRKY72, and DGK5, respectively; see the table legend for full names of proteins), regulation of growth and development (Table S5, ON_4071, ON_4867, and ON_5499, encoding CYCA2;4, TFL1, and XCP1, respectively), regulation of organelle functions and senescence (Table S5, ON_5701 and ON_4729 for encoding and a UQCRX-like family protein), and embryogenesis (Table S5, ON_4633/KPL).

We suggest that these genes, based on their lineage-specific *tr-d* events leading to additional gene copies that are expressed and also encode complete ORFs, may have functions in salt adaptation yet unknown in the salt-sensitive model *A. thaliana*, or their functions, either conserved or modified, may regulate a strategic decision-making process in growth and development in avoiding or alleviating salt stresses (Julkowska and

Testerink 2015) in these two extremophyte species. Additional tissue- and stress response-specific expression data and functional studies using genetic and transgenic methods will be able to refine and confirm these hypotheses.

S8. *tr-d* events shared by extremophyte lineages and signatures of a convergent evolution

Gene copy number variations through *tr-d* events shared exclusively by the two extremophytes may represent a signature of convergent evolution in two separate lineages that show salt-adaptive traits (Orsini et al. 2010). The OrthNet pipeline identified two subsets of *tr-d* events shared by the two extremophytes, of which the complete list is presented in Table S6. Three parallel *tr-d* events independently occurred in both *E. salsugineum* and *S. parvula* (Table S6, "Ind-parallel") involved loci encoding well-known salt stress signal transducers in the model plant *A. thaliana* (Table S6, ABI1, CDPK1, and 5PTASE11, see the table legend for full names). However, none of these events resulted in additional gene copies with complete ORFs and expression evidence in both genomes (Table S6). The other subset of shared *tr-d* events most likely took place in a Lineage II ancestor before the divergence of *E. salsugineum* and *S. parvula*, based on the fact that the Tr copies in both genomes showed co-linearity as depicted in Fig. 5A. Subsequent deletion or truncation of all duplicated Tr copies in *S. irio* made these tr-d events unique to the two extremophytes (Table S6, "Tr-*cl*"). This subset included two OrthNets with *tr-d*, encoding transcription factors of unknown functions (Table S6, ON_170-3 and ON_3847) (Cheng et al. 2017). In both cases, Tr copy loci included complete ORFs with expression evidence in both extremophyte species. Additionally, a well-known salt stress signaling gene locus encoding the Calcineurin B-Like protein 10 (Quan et al. 2007; Ren et al. 2013) showed *tr-d* in both *E. salsugineum* and *S. parvula* with complete ORFs in Tr copy loci. However, expression was detected only for the two *S. parvula* Tr copies (Table S6, ON_2430/CBL10).

The paucity of copy number enhancement with complete ORFs through shared parallel *tr-d* events suggests that the adaptation path was distinct and unique to each extremophyte species. While being the two most salt-tolerant among tested Brassicaceae species (Orsini et al. 2010), the natural habitats of these extremophytes do present distinct combinations of challenges for each species (Helvaci et al. 2004; Inan 2004; Oh et al. 2014). The increased copy numbers of the two transcription factors found among the "*Tr-cl*" subset however, present a good set of candidates for further genetic and transgenic studies, to determine their possible contribution to the extremophytes' responses to high salinity, the common stress component of their habitats.

S9. Flexibility of the CLfinder-OrthNet pipeline and future directions

The CLfinder-OrthNet pipeline provides a flexible tool to identify co-linearity among orthologs in multiple closely related genomes and organize them into networks enabling a search based on evolutionary contexts for lineage- or lineages-specific gene duplication and transposition events. The input for paralog clusters, used for identification of tandem duplication events in each genome (Fig. S2, "Input 2"), can be derived from any gene clustering method, with a user-defined parameter for the maximum loci difference

between tandem duplicates. Likewise, users can choose the method and criteria for inter-species comparisons to determine best-hit pairs (Fig. S2, “Input 3”). In this study, we aimed to trace the *tr-d* events including truncated ORFs. Hence we considered all best-hits detected by BLASTN (Camacho et al. 2009) among coding sequences with an e-value cutoff ($e<10^{-5}$) and no additional criteria. Users interested more on identifying conserved protein functions can apply additional filters, such as conserved ORF lengths, deduced amino acid similarities, and conserved peptide domain structures, to ensure detection of best-hit pairs among functional ORFs. Finally, the genome annotation input (Fig. S2, “Input 1”) can include non-protein-coding features such as putative enhancers identified as intergenic DNase Hypersensitive regions (Zhu et al. 2015), miRNA genes, or any other conserved regulatory feature that can be traced among multiple closely related genomes which often share substantial sequence similarity over intergenic regions (Haudry et al. 2013).

REFERENCES

Ali A, Raddatz N, Aman R, Kim S, Park HC, Jan M, Baek D, Khan IU, Oh D-H, Lee SY, et al. 2016. A Single Amino Acid Substitution in the Sodium Transporter HKT1 Associated with Plant Salt Tolerance. Plant Physiol 171:pp.00569.2016.

Ali Z, Park HC, Ali A, Oh D-HD-H, Aman R, Kropornicka A, Hong H, Choi W, Chung WS, Kim W-YW-Y, et al. 2012. TsHKT1;2, a HKT1 Homolog from the Extremophile Arabidopsis Relative *Thellungiella salsuginea*, Shows K^+ Specificity in the Presence of NaCl. Plant Physiol 158:1463–1474.

Amtmann A. 2009. Learning from evolution: *Thellungiella* generates new knowledge on essential and critical components of abiotic stress tolerance in plants. Mol Plant 2:3–12.

Baidouri M El, Panaud O. 2013. Comparative genomic paleontology across plant kingdom reveals the dynamics of TE-driven genome evolution. Genome Biol Evol 5:954–965.

Camacho C, Coulouris G, Avagyan V, Ma N, Papadopoulos J, Bealer K, Madden TL. 2009. BLAST+: architecture and applications. BMC Bioinformatics 10:421.

Chen F, Mackey AJ, Stoeckert CJ, Roos DS. 2006. OrthoMCL-DB: querying a comprehensive multi-species collection of ortholog groups. Nucleic Acids Res 34:D363–D368.

Cheng C-Y, Krishnakumar V, Chan AP, Thibaud-Nissen F, Schobel S, Town CD. 2017. Araport11: a complete reannotation of the Arabidopsis thaliana reference genome. Plant J 89:789–804.

Dassanayake M, Oh D-H, Hong H, Bohnert HJ, Cheeseman JM. 2011. Transcription strength and halophytic lifestyle. Trends Plant Sci 16:1–3.

Emms DM, Kelly S. 2015. OrthoFinder: solving fundamental biases in whole genome comparisons dramatically improves orthogroup inference accuracy. Genome Biol 16:157.

Freeling M. 2009. Bias in plant gene content following different sorts of duplication: tandem, whole-genome, segmental, or by transposition. Annu Rev Plant Biol 60:433–453.

Gong Q, Li P, Ma S, Indu Rupassara S, Bohnert HJ. 2005. Salinity stress adaptation competence in the extremophile Thellungiella halophila in comparison with its relative Arabidopsis thaliana. Plant J 44:826–839.

Haudry A, Platts AAE, Vello E, Hoen DRD, Leclercq M, Williamson RJ, Forczek E, Joly-Lopez Z, Steffen JG, Hazzouri KM, et al. 2013. An atlas of over 90,000 conserved noncoding sequences provides insight into crucifer regulatory regions. Nat Genet 45:891–898.

Helvaci C, Mordogan H, Çolak M, Gündoğan I. 2004. Presence and Distribution of Lithium in Borate Deposits and Some Recent Lake Waters of West-Central Turkey. Int Geol Rev 46:177–190.

Hu TTTT, Pattyn P, Bakker EGEG, Cao J, Cheng JJF, Clark RM, Fahlgren N, Fawcett JA, Grimwood J, Gundlach H, et al. 2011. The Arabidopsis lyrata genome sequence and the basis of rapid genome size change. Nat Genet 43:476–481.

Inan G. 2004. Salt Cress. A Halophyte and Cryophyte Arabidopsis Relative Model System and Its Applicability to Molecular Genetic Analyses of Growth and Development of Extremophiles. Plant Physiol 135:1718–1737.

Julkowska MM, Testerink C. 2015. Tuning plant signaling and growth to survive salt. Trends Plant Sci:1–9.

Lysak MA, Berr A, Pecinka A, Schmidt R, McBreen K, Schubert I. 2006. Mechanisms of chromosome number reduction in Arabidopsis thaliana and related Brassicaceae species. Proc Natl Acad Sci USA 103:5224–5229.

Oh D-H, Dassanayake M, Haas JS, Kropornika A, Wright C, D'Urzo MP, Hong H, Ali S, Hernandez A, Lambert GM, et al. 2010. Genome structures and halophyte-specific gene expression of the extremophile *Thellungiella parvula* in comparison with *Thellungiella salsuginea* (*Thellungiella halophila*) and Arabidopsis. Plant Physiol 154:1040–1052.

Oh D-H, Hong H, Lee SY, Yun D-J, Bohnert HJ, Dassanayake M. 2014. Genome Structures and Transcriptomes Signify Niche Adaptation for the Multiple-Ion-Tolerant Extremophyte Schrenkiella parvula. Plant Physiol 164:2123–2138.

Oh D-H, Leidi E, Zhang Q, Hwang S-M, Li Y, Quintero FJ, Jiang X, D'Urzo MP, Lee SY, Zhao Y, et al. 2009. Loss of Halophytism by Interference with SOS1 Expression. Plant Physiol 151:210–222.

Orsini F, D'Urzo MP, Inan G, Serra S, Oh DH, Mickelbart M V., Consiglio F, Li X, Jeong JC, Yun DJ, et al. 2010. A comparative study of salt tolerance parameters in 11 wild relatives of Arabidopsis thaliana. J Exp Bot 61:3787–3798.

Quan R, Lin H, Mendoza I, Zhang Y, Cao W, Yang Y, Shang M, Chen S, Pardo JM, Guo Y. 2007. SCABP8/CBL10, a putative calcium sensor, interacts with the protein kinase SOS2 to protect Arabidopsis shoots from salt stress. Plant Cell 19:1415–1431.

Ren XL, Qi GN, Feng HQ, Zhao S, Zhao SS, Wang Y, Wu WH. 2013. Calcineurin B-like protein CBL10 directly interacts with AKT1 and modulates K+ homeostasis in Arabidopsis. Plant J 74:258–266.

Wicker TM, Buchmann JP, Keller B. 2010. Patching gaps in plant genomes results in gene movement and erosion of colinearity. Genome Res 20:1229–1237.

Woodhouse MR, Pedersen B, Freeling M. 2010. Transposed genes in Arabidopsis are often associated with flanking repeats. PLoS Genet 6:26.

Wu H-J, Zhang Z, Wang J-Y, Oh D-H, Dassanayake M, Liu B, Huang Q, Sun H-X, Xia R, Wu Y, et al. 2012. Insights into salt tolerance from the genome of *Thellungiella salsuginea*. Proc Natl Acad Sci USA 109:12219–12224.

Zhu B, Zhang W, Zhang T, Liu B, Jiang J. 2015. Genome-Wide Prediction and Validation of Intergenic Enhancers in Arabidopsis Using Open Chromatin Signatures. Plant Cell 27:2415–2426.
